# Development and validation of the C-reactive protein–triglyceride-glucose index for predicting short- and long-term mortality in critically ill patients with coronary artery disease: a multicenter cohort study

**DOI:** 10.3389/fcvm.2026.1763569

**Published:** 2026-05-13

**Authors:** Jian Yang, Yilin Xia, Weixi Guo, Liqun Wu, Xusheng Wu, Bei Li

**Affiliations:** 1 Department of Biomedical Informatics, School of Life Sciences, Central South University, Changsha, China; 2 Shenzhen Health Development Research and Data Management Center, Shenzhen, China

**Keywords:** coronary artery disease, c-reactive protein-triglyceride-glucose index, inflammation, insulin resistance, machine learning

## Abstract

**Background:**

The C-reactive protein–triglyceride glucose index (CTI) has been proposed as a novel biomarker of insulin resistance and inflammation, but its association with mortality in critically ill patients with coronary artery disease (CAD) remains unclear. This study aimed to evaluate the associations between the CTI and both short- and long-term all-cause mortality and to assess the predictive value of this index.

**Methods:**

Patients with CAD were identified from the MIMIC-IV database and divided into internal training and testing cohorts, while an external validation cohort was derived from the eICU-CRD and Shenzhen Regional Health Information Platform (SRHIP) database. Based on the optimal CTI cut-off values, patients were grouped into three categories. The primary outcomes were short-term (30-day) and long-term (365-day) all-cause mortality. Associations between the CTI and mortality were examined using Kaplan–Meier curves, restricted cubic spline regression, and Cox proportional hazards models. Subgroup, mediation and sensitivity analyses tested result robustness. The CTI was further compared with other single predictors, and six machine learning (ML) models were built to assess its predictive performance. Finally, the SHapley Additive exPlanations (SHAP) analysis identified feature contributions, and a user-friendly web application was developed.

**Results:**

The primary cohort included 1,561 patients, and two external validation cohorts included 242 and 105 patients from the eICU-CRD and SRHIP databases. High CTI values were significantly associated with increased short- and long-term mortality, demonstrating a nonlinear dose–response relationship. The CTI exhibited particularly high predictive value for short-term outcomes. The incorporation of the CTI into ML models notably improved the predictive performance, and this improvement was confirmed in the external validation cohort.

**Conclusions:**

The CTI was identified as an independent predictor of short- and long-term mortality in critically ill patients with CAD, with a particularly high predictive value for short-term risk stratification. Integrating the CTI into predictive models significantly increased the prognostic accuracy.

## 1 Introduction

Cardiovascular disease (CVD) remains the leading cause of death worldwide. CVD accounts for nearly 20 million deaths annually, representing approximately one-third of all deaths, with more than three-quarters occurring in low- and middle-income countries (1). Coronary artery disease (CAD) is the most prevalent type of CVD, with an estimated mortality rate of 108.8 per 100,000 people (2). Its incidence and mortality have greatly increased in most countries and regions (3), making it a major global health problem. Comprehensive research is therefore essential to identify key risk factors for CAD and to develop effective management and treatment strategies.

Insulin resistance (IR) is characterized by reduced sensitivity to insulin, leading to impaired glucose use and metabolic disorders. As a key pathological mechanism of metabolic syndrome and atherosclerosis, IR promotes inflammation, oxidative stress, and endothelial dysfunction, ultimately accelerating the progression of coronary atherosclerosis and increasing the risk of CAD (4–6). In epidemiological studies and clinical practice, the triglyceride–glucose (TyG) index, has been widely recognized as a reliable surrogate marker for IR due to its simplicity and strong correlations with hyperinsulinemia and insulin sensitivity, and it has been validated across diverse populations and regions (7–9). Accumulating evidence indicates that an elevated TyG index is closely associated with an increased risk of CAD, greater severity of coronary lesions, and worse clinical outcomes (10). Moreover, the role of inflammation in driving atherosclerosis and CAD progression has been well established. C-reactive protein (CRP), a nonspecific marker of systemic inflammation, not only reflects the residual inflammatory risk but is also independently associated with major adverse cardiovascular events (MACEs), cardiovascular mortality, and all-cause mortality in patients with CAD. Further studies have emphasized that both chronic inflammation and atherosclerotic dyslipidemia should be jointly assessed and managed in the primary prevention of CVD (11, 12). Therefore, the development of a composite indicator that simultaneously reflects IR and the inflammatory status to predict cardiovascular risk holds significant clinical value. In this context, Ruan et al. (13) first proposed integrating CRP levels with the TyG index to establish the C-reactive protein–triglyceride-glucose index (CTI), which is designed to comprehensively assess both inflammation and IR. This index has shown good predictive ability in various clinical settings, including in determining the cancer cachexia prognosis, cancer mortality in the general population, and the risk of developing depression (14, 15). However, the association between CTI and CAD, particularly its ability to predict short- and long-term all-cause mortality in critically ill patients with CAD, remains unclear, as existing studies have largely focused on general populations or metabolism-related cohorts. For example, a cross-sectional analysis of National Health and Nutrition Examination Survey (NHANES) data revealed that individuals in the highest CTI quartile exhibited approximately twice the risk of coronary heart disease (CHD) compared with those in the lowest quartile (16). Ou et al. used data from the China Health and Retirement Longitudinal Study (CHARLS) cohort and reported that CTI could capture cardiovascular events and all-cause mortality in individuals with stage 0–3 cardiometabolic syndrome (17). Other studies have shown that CTI is positively associated with incident CHD, particularly among individuals who are metabolically unhealthy but exhibit a normal weight, and that CTI may increase the predictive accuracy when it is combined with other inflammatory or nutritional markers (18). Nevertheless, none of these studies have focused specifically on critically ill patients with CAD, and evidence regarding the relationships between CTI and short- and long-term all-cause mortality remains limited.

In recent years, machine learning (ML) has emerged as an important approach for developing clinical prediction models. The advantage of ML lies in its ability to automatically learn patterns from high-dimensional, nonlinear, and complex interactions, often achieving superior predictive performance to traditional statistical models. For example, an ML model incorporating single-photon emission computed tomography imaging features and demographic variables predicted MACEs and all-cause mortality in CAD patients, achieving a sensitivity and specificity greater than 65% and outperforming logistic regression (19). Huang et al. (20) applied the extreme gradient boosting (XGBoost) algorithm in CAD diagnosis, and achieved an accuracy greater than 85%. In addition, Li et al. (21) developed a gradient boosting machine (GBM) model for elderly Chinese patients with CAD presenting with impaired glucose tolerance or diabetes, which showed optimal performance in predicting 1-year mortality. Although progress has been made in the application of ML to determining the CAD prognosis, research evaluating the role of CTI in the prognosis of CAD patients in the ICU is lacking, in particular, studies incorporating CTI into the ML framework to predict short- and long-term all-cause mortality in critically ill patients with CAD are lacking.

Traditional etiological statistical models provide intuitive hazard ratios (HRs) and time-to-event curves with strong clinical interpretability, but they are limited in capturing complex nonlinear relationships. In contrast, ML is well suited for handling high-dimensional features and increasing predictive accuracy. However, its “black-box” nature often limits interpretability. Therefore, this study adopted a dual validation strategy that combined traditional etiological statistical methods with ML. Conventional survival analysis methods were applied to evaluate the associations between the CTI and both short- and long-term all-cause mortality. In parallel, the CTI was integrated with key clinical features to develop and compare six ML models for more precise mortality risk stratification in critically ill patients with CAD. Although etiological analysis and predictive modeling differ in their objectives, applications, and methodological approaches, they can play complementary roles in prognostic research and together form a progressive chain of evidence (22–24). Moreover, the combined use of these two approaches in studies of clinical outcomes in critically ill patients is becoming increasingly common, supporting the feasibility and value of linking association-level evidence with application-oriented predictions (25, 26).

## 2 Methods

### 2.1 Study design

**Figure 1:**
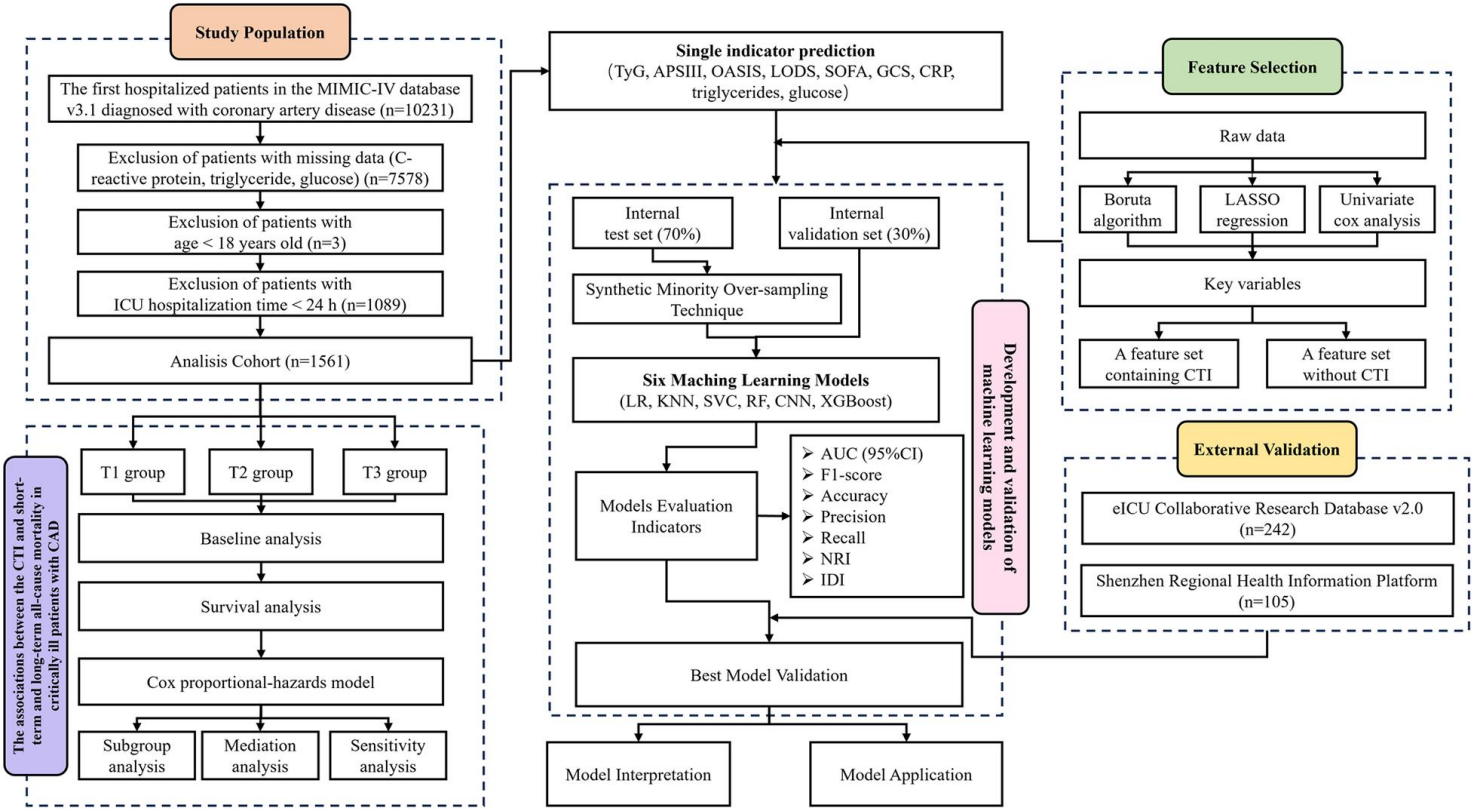
Flowchart of the study design process.

The overall study design is shown in Figure 1. First, based on predefined inclusion and exclusion criteria, a total of 1,561 patients with coronary artery disease were identified from the Medical Information Mart for Intensive Care IV (MIMIC-IV) database to form the analytic cohort. The cohort was then stratified into three groups according to CTI levels. Kaplan–Meier survival analysis, restricted cubic spline (RCS) modeling, and multivariable Cox proportional hazards regression were performed to evaluate the associations between CTI and short- and long-term all-cause mortality, while subgroup and sensitivity analyses were conducted to assess the robustness of the findings. Second, to systematically evaluate the predictive value of CTI for mortality risk, its predictive performance was first compared with that of other individual biomarkers. Guided by feature selection, six ML models were subsequently developed and internally validated. Model performance was assessed using the area under the curve (AUC), F1-score, accuracy, precision, recall, net reclassification improvement (NRI), and integrated discrimination improvement (IDI). External validation was then performed using two independent cohorts: a same-region validation cohort derived from the eICU Collaborative Research Database (eICU-CRD) and a cross-region validation cohort obtained from the Shenzhen Regional Health Information Platform (SRHIP). Finally, SHapley Additive exPlanations (SHAP) was applied to interpret feature contributions in the optimal model, and a user-friendly web application was developed to enhance clinical applicability.

### 2.2 Data source

Data for this study were obtained from three databases: MIMIC-IV (version 3.1) (27), eICU-CRD (version 2.0) (28), and SRHIP. MIMIC-IV contains deidentified clinical data from approximately 190,000 patients and 450,000 hospital admissions at BIDMC between 2008 and 2022, including demographics, laboratory tests, vital signs, medications, diagnoses, procedures, and outcomes. The eICU-CRD contains deidentified clinical data from 139,367 patients and more than 200,000 ICU admissions across 208 hospitals in the continental United States between 2014 and 2015. There is no overlap in contributing hospitals between the MIMIC-IV and eICU-CRD. The dataset covers demographics, diagnoses, laboratory results, and treatment information. The SRHIP aggregates health records from Shenzhen, China, integrating data from 75 public hospitals, 106 private hospitals, and over 1,600 community health service centers. It functions as a comprehensive health data center, housing over 40 million electronic health archives and billions of service records. In this study, the MIMIC-IV was primarily used for original data analyses and for internal training and validation of the ML models. The eICU-CRD was used as a same-region external validation set for the ML models to evaluate generalizability, with the SRHIP serving as the cross-region external validation cohort.

In accordance with the data usage protocols for MIMIC-IV and eICU-CRD, one of the authors completed the required human subjects research training (Record ID: 71784813) and signed the Data Use Agreements (DUA). The use of these two databases was approved by the Institutional Review Boards (IRBs) of the Beth Israel Deaconess Medical Center (BIDMC) and the Philips eICU Research Institute. For the SRHIP data, this study was approved by the Ethics Committee of the Shenzhen Health Development Research and Data Management Center (registration number: 2025009). Due to the retrospective nature of the study and the use of de-identified or anonymized data, the requirement for informed consent was waived for all three databases.

The study also adhered to the Strengthening the Reporting of Observational Studies in Epidemiology (STROBE) guidelines (29) and the Declaration of Helsinki.

### 2.3 Study population

Patients with a first ICU admission for CAD were identified from the MIMIC-IV, eICU-CRD, and SRHIP databases using the International Classification of Diseases (ICD)-9 and ICD-10 codes. The following exclusion criteria were applied across all databases: (1) age <18 years at ICU admission; (2) ICU stay <24 h; (3) multiple ICU admissions for CAD, with only the first admission included; and (4) missing data on CRP, triglyceride, and fasting glucose. In total, 1,561 patients from MIMIC-IV, 242 from eICU-CRD, and 105 from SRHIP were included in the final analysis.

### 2.4 Data extraction

Clinical data were extracted using structured query language (SQL) via PostgreSQL (version 8.7.0) and Navicat Premium (version 16.3.2). Based on expert consultation and a literature review (30), clinical features were extracted across seven categories: demographics, vital signs, comorbidities, laboratory indicators, clinical treatments, scoring systems, and clinical outcomes. The extracted data included the first recorded values of all clinical variables within 24 h after the first ICU admission, including laboratory measurements such as C-reactive protein, triglyceride, and glucose. These variables were collected prior to major interventions, such as PCI or CABG, and thereby reflected the patients' baseline clinical status at ICU admission. A detailed list of these variables is presented in Table 1. Missing data were imputed using the random forest method to reduce potential bias.

**Table 1:** Extraction of information for the variables.

Items	Composition
Demographics	Age, gender, BMI, marriage, race
Vital signs	Heart rate, SBP, DBP, MBP, Resp rate, temperature, Spo2
Comorbidities	Hypertension, diabetes, AHF, hyperlipidemia, obesity, CKD, AMI
Laboratory indicators	CRP, hematocrit, platelet, bun, creatinine, potassium, sodium, magnesium, PT, INR, RDW, bicarbonate, eGFR, triglyceride, fasting blood glucose, WBC, RBC, ALT, ALP, hemoglobin, total bilirubin, free calcium, phosphate, chloride, lymphocytes, CTI
Clinical treatment	Warfarin, statin, beta blocker, NOAC, antiplatelet, metformin, insulin, vasopressin, octreotide, ventilated, RRT
Scoring system	APSIII, OASIS, LODS, SOFA, GCS
Clinical outcomes	Los Icu, Los Hospital, 30-day mortality, 90-day mortality, 180-day mortality, 365-day mortality

### 2.5 Definitions of CTI and study endpoint

In this study, the CTI was calculated as follows:

$$\begin{array}{c} CTI=0.412\times lnCRP(mg/L)+ln\frac{TG(mg/dl)\times FBG(mg/dl)}{2} \end{array}$$ (1)

In Equation 1, CRP represents C-reactive protein, TG represents triglyceride, and FBG represents fasting blood glucose. The primary endpoints of this study were all-cause mortality in patients with CAD at 30-day (short term) and 365-day (long term), and the secondary endpoints included all-cause mortality at 90-day and 180-day.

### 2.6 Statistical analysis

Continuous variables with a normal distribution are presented as the means ± standard deviations (SDs), whereas those with a nonnormal distribution are presented as the medians and interquartile ranges (IQRs). Normality was assessed using the Shapiro–Wilk test. The differences in continuous variables between groups were assessed using Student's t test or the Kruskal–Wallis test, depending on the distribution. Categorical variables are presented as counts (percentages), and differences between groups were analyzed using the chi-square test or Fisher's exact test.

X-tile software (version 3.6.1) is widely used in medical and epidemiological research to identify optimal cut-off points for continuous survival-related variables. In this study, X-tile was chosen to determine the optimal cut-off point of the CTI using 30-day mortality as the endpoint. Patients were then stratified into three groups according to the optimal cut-off points. Kaplan–Meier survival curves were created to illustrate cumulative all-cause mortality across CTI groups, and the log-rank test was applied to assess statistical significance. Multivariable Cox proportional hazards models were constructed to further examine the association between CTI levels and all-cause mortality in critically ill patients with CAD across different follow-up periods. Potential confounders were selected according to three criteria: (1) variables with variance inflation factor (VIF) <5; (2) variables with *p* < 0.05 in the univariate analyses; and (3) factors previously recognized in the literature or clinical practice as important prognostic variables. Three progressively adjusted models were constructed: Model 1, unadjusted; Model 2, adjusted for Hyperlipidemia, Hypertension, Diabetes, Gender, Spo2, BMI, HR, RR, Age and SBP; and Model 3, further adjusted for Beta Blocker, Statin, Antiplatelet, Metformin, Vasopressin, Warfarin, Insulin, Free Calcium, ALP, ALT, WBC, Platelet, Lymphocytes, Bicarbonate, Sodium, Phosphate, Los Hospital, OASIS, LODS, APSIII and Bun.

Subgroup analyses were performed according to age (<65 or ≥65 years), sex, race, hyperlipidemia, diabetes, acute heart failure, hypertension, obesity, chronic kidney disease, and acute myocardial infarction to assess the consistency of the predictive value of CTI across patients with different clinical characteristics. In addition, a bootstrap-based mediation analysis was conducted to investigate the potential mediating effects of five illness severity scores, namely APSIII, OASIS, LODS, SOFA, and GCS, in the association between CTI and mortality risk at each time point.

Three sensitivity analyses were conducted to assess the robustness of the findings: (1) the association between CTI, which was treated as a continuous variable, and mortality risk was examined using Cox proportional hazards models; (2) E-values were calculated for the Cox models to quantify potential bias from unmeasured confounders (31); and (3) logistic regression models were used to confirm the association between CTI and mortality risk.

Subsequently, receiver operating characteristic (ROC) curves were analyzed to evaluate the predictive performance of ten indicators (TyG, APSIII, OASIS, LODS, SOFA, GCS, CRP, triglyceride, glucose, and CTI) for mortality at 30-, 90-, 180-, and 365-day follow-up. Predictive performance was assessed on the basis of AUC, sensitivity, and specificity.

Finally, ML models were developed to predict all-cause mortality in critically ill patients with CAD and were validated using multicenter data. The analytic cohort from MIMIC-IV was randomly divided into a training set and an internal validation set at a 7:3 ratio. In the training set, feature selection was performed using Boruta, LASSO regression, and univariate Cox regression. Six ML models were developed: logistic regression (LR), k-nearest neighbor (KNN), support vector classifier (SVC), random forest (RF), eXtreme Gradient Boosting (XGBoost), and convolutional neural network (CNN). Each model was trained using either the baseline features alone or the baseline features plus CTI. Hyperparameters were optimized using fivefold cross-validation and grid search, and class imbalance in the training set was corrected using the Synthetic Minority Over-sampling Technique (SMOTE). Model performance was evaluated and compared in the internal validation set using the AUC, F1 score, accuracy, precision, recall, NRI, and IDI. The best-performing model was externally validated in the eICU-CRD and SRHIP cohorts. Its generalizability was assessed by constructing ROC curves, calibration curves, and decision curve analysis (DCA). To improve interpretability, SHAP values were calculated to quantify the contributions of individual features to model predictions. A web-based platform incorporating the optimal model was developed to support clinical application.

All the statistical analyses were performed in Python (version 3.9.7) and R (version 4.4.0), and a two-sided *p* value < 0.05 was considered statistically significant.

## 3 Results

### 3.1 Baseline characteristics

**Figure 2:**
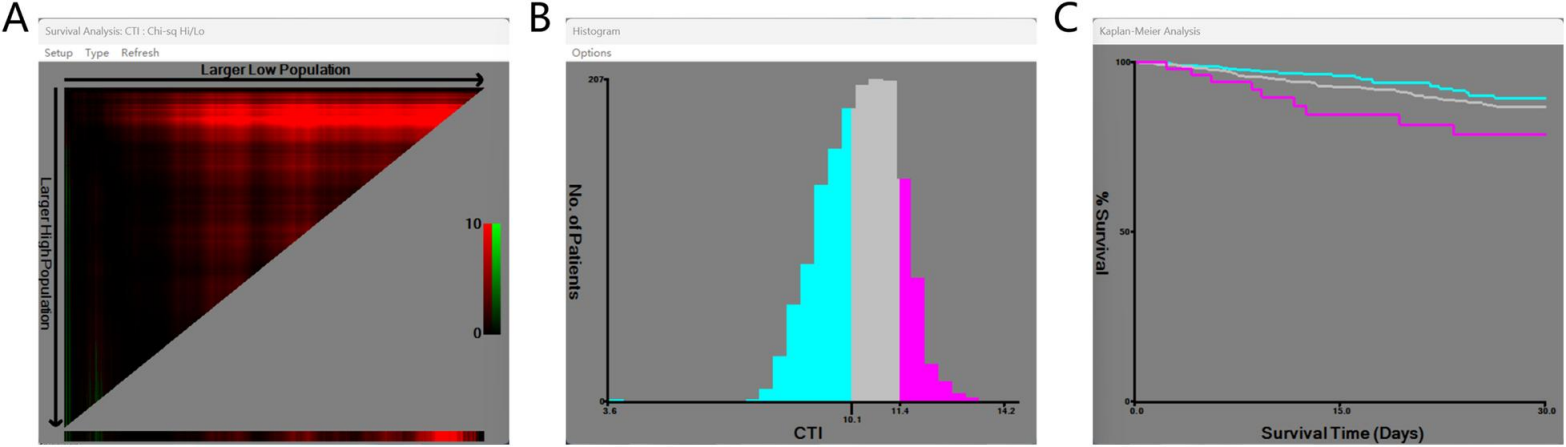
Chart showing the selection of the optimal cut-off point selection.

A total of 1,561 critically ill patients who were diagnosed with critically ill CAD were included from the MIMIC-IV database. Via the use of X-tile software, the optimal CTI cut-off values were identified as 10.1 and 11.4 based on 30-day all-cause mortality after ICU admission (Figure 2). Patients were accordingly stratified into three groups: low CTI (T1, <10.1), intermediate CTI (T2, 10.1–11.4), and high CTI (T3, >11.4). The baseline characteristics of the three groups are presented in Supplementary Table S1. In the overall cohort, 1,015 patients (65.02%) were male, the median age was approximately 70 years, and 1,032 patients (66.11%) were white. During the 365-day follow-up period, 275 patients (17.62%) experienced all-cause mortality. Compared with patients in the low- and intermediate-CTI groups, patients in the high-CTI group were older, more likely to be male, and had higher BMI, heart rate, and respiratory rate. They also had higher incidences of diabetes, acute heart failure, obesity, and chronic kidney disease, as well as significantly higher APSIII and OASIS scores. In terms of laboratory indicators, patients in the high-CTI group exhibited higher levels of platelets, blood urea nitrogen, creatinine, red blood cells, white blood cells, alanine aminotransferase, and alkaline phosphatase. However, they had lower use rates of warfarin, beta-blockers, and antiplatelet agents. Moreover, higher CTI values were associated with increased all-cause mortality at 30-day (4.78% vs. 7.16% vs. 13.08%, *P* < 0.001), 90-day (6.78% vs. 10.60% vs. 18.22%, *P* < 0.001), 180-day (8.94% vs. 14.61% vs. 23.36%, *P* < 0.001), and 365-day (12.17% vs. 19.20% vs. 28.97%, *P* < 0.001). In addition, the ICU length of stay [2.00 (1.27–3.21) vs. 2.33 (1.45–4.13) vs. 3.29 (1.77–5.91), *P* < 0.001] and overall hospitalization duration [7.06 (4.97–10.93) vs. 8.77 (5.72–13.85) vs. 10.44 (6.65–17.13), *P* < 0.001] increased with increasing CTI. The baseline characteristics of the eICU-CRD and the SRHIP database were presented in Supplementary Tables S2, S3, respectively.

### 3.2 Survival analysis

**Figure 3:**
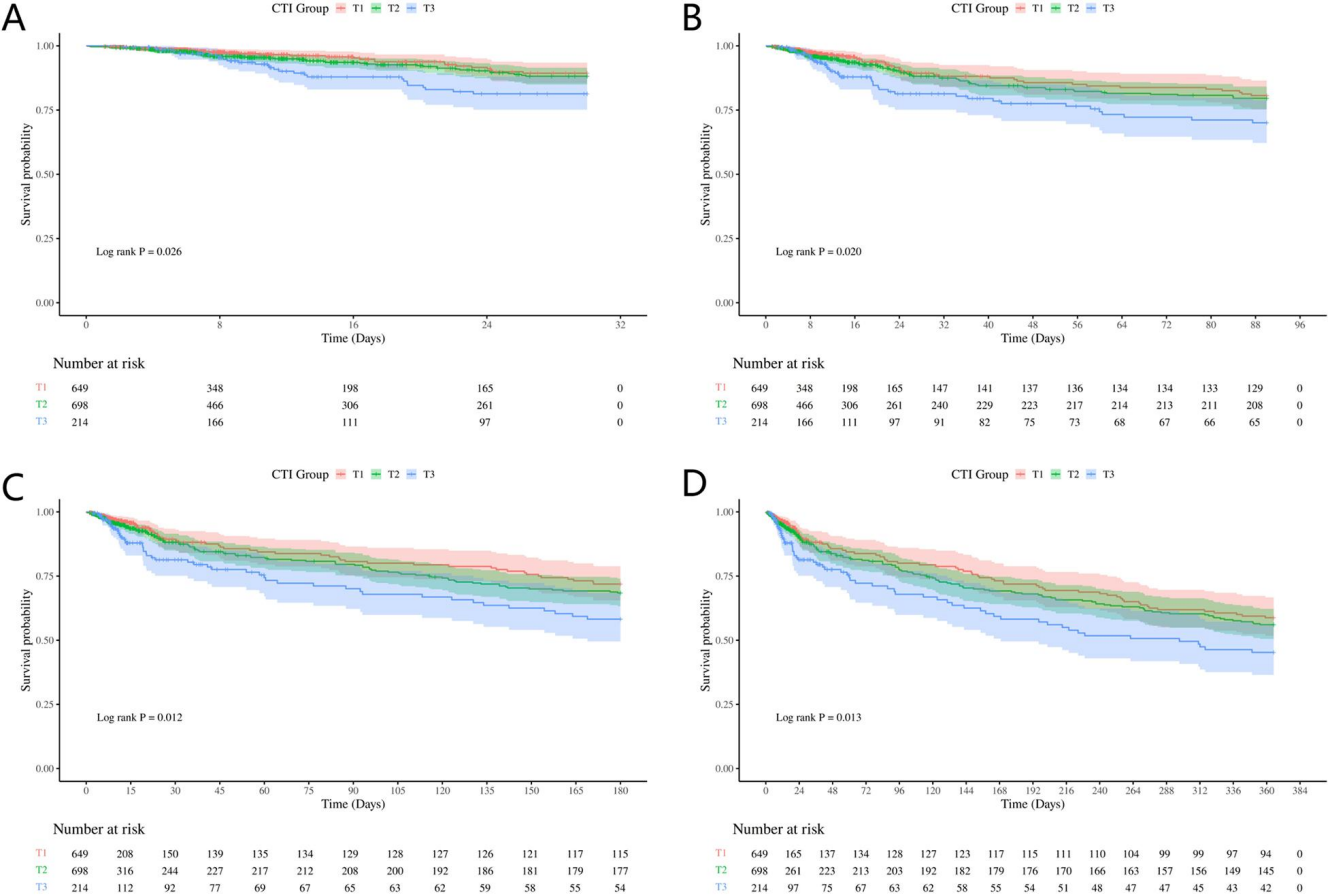
Kaplan–meier survival curves. **(A)** Comparison of all-cause mortality between groups at 30-day. **(B)** Comparison of all-cause mortality between groups at 90-day. **(C)** Comparison of all-cause mortality between groups at 180-day. **(D)** Comparison of all-cause mortality between groups at 365-day.

A Kaplan–Meier survival analysis was performed to evaluate all-cause mortality across CTI groups at different follow-up time points (Figure 3). The log-rank test indicated significant differences in all-cause mortality among the three groups at 30, 90, 180, and 365-day (*P* = 0.026, 0.020, 0.012, and 0.013, respectively). Throughout follow-up, the high-CTI group (T3) consistently had the highest all-cause mortality compared with the other groups, and the survival differences became more pronounced over time.

To further compare the survival stratification performance of CTI with that of its individual components, additional Kaplan–Meier analyses were performed for CRP and TyG (Supplementary Figure S1). The results showed that, although CRP was associated with significant survival differences at some follow-up time points, its stratification performance was less consistent than that of CTI, whereas TyG did not demonstrate statistically significant survival separation across the follow-up periods. These findings suggest that CTI may provide more stable survival stratification than either CRP or TyG alone.

### 3.3 The associations between CTI and short-term and long-term all-cause mortality in critically ill patients with CAD

Three Cox proportional hazards models were established to examine the associations and independent effects of the CTI on the short-term and long-term survival of critically ill patients with CAD.

Prior to constructing the risk models, covariates were identified. Variables with a VIF >5 were excluded to avoid multicollinearity, and 54 variables were initially retained (Supplementary Table S4). Based on the univariate Cox regression analysis results (Supplementary Table S5), previous studies, and clinical expertise, 32 covariates were ultimately selected for multivariable adjustment, including Hyperlipidemia, Hypertension, Diabetes, Gender, Spo2, BMI, HR, RR, Age, SBP, Beta Blocker, Statin, Antiplatelet, Metformin, Vasopressin, Warfarin, Insulin, Free Calcium, ALP, ALT, WBC, Platelet, Lymphocytes, Bicarbonate, Sodium, Phosphate, Los Hospital, OASIS, LODS, SBP, APSIII, and Bun.

The results of the multivariable Cox regression analyses are presented in Table 2. When the low CTI group (T1) was used as a reference, the HRs for the intermediate group (T2) were not statistically significant at any time point (all *P* > 0.05). In contrast, the mortality risk was significantly higher in the high CTI group (T3) than in the T1 group at 30-, 90-, 180-, and 365-day. In unadjusted Model 1, the HRs for T3 were 1.97 (95% CI: 1.18–3.31, *P* < 0.001), 1.79 (95% CI: 1.16–2.76, *P* < 0.001), 1.75 (95% CI: 1.20–2.56, *P* = 0.003), and 1.62 (95% CI: 1.16–2.27, *P* = 0.004) at 30-, 90-, 180-, and 365-day, respectively. In Model 2, which was adjusted for age and sex, the HRs for T3 increased to 2.36 (95% CI: 1.36–4.09), 2.23 (95% CI: 1.40–3.54), 2.17 (95% CI: 1.45–3.26), and 1.88 (95% CI: 1.32–2.68) (all *P* < 0.001). In the fully adjusted Model 3, the HRs for T3 slightly decreased to 2.14, 1.84, 1.79, and 1.40 but remained statistically significant (all *P* < 0.001), indicating that the CTI was independently associated with increased mortality risk. Moreover, the *P* for trend was significant in all models at all time points (all *P* < 0.05), supporting a dose–response relationship between higher CTI levels and mortality risk and underscoring the potential of CTI as an independent prognostic predictor.

**Table 2:** Multivariate Cox proportional hazards model of all-cause mortality at each time point.

Outcome	Model 1 HR (95%CI)	*P*	Model 2 HR (95%CI)	*P*	Model 3 HR (95%CI)	*P*
30d						
T1	1.00 (Reference)		1.00 (Reference)		1.00 (Reference)	
T2	1.22 (0.77–1.92)	0.392	1.33 (0.84–2.10)	0.228	1.41 (0.88–2.25)	0.148
T3	1.97 (1.18–3.31)	0.01	2.36 (1.36–4.09)	0.002	2.14 (1.21–3.81)	<.001
*P* for trend	1.40 (1.07–1.83)	0.013	1.59 (1.21–2.10)	<.001	1.46 (1.10–1.95)	0.009
90d						
T1	1.00 (Reference)		1.00 (Reference)		1.00 (Reference)	
T2	1.15 (0.79–1.68)	0.453	1.24 (0.85–1.81)	0.27	1.13 (0.76–1.68)	0.543
T3	1.79 (1.16–2.76)	0.008	2.23 (1.40–3.54)	<.001	1.84 (1.11–3.05)	0.017
*P* for trend	1.33 (1.07–1.67)	0.011	1.52 (1.20–1.91)	<.001	1.34 (1.06–1.71)	0.016
180d						
T1	1.00 (Reference)		1.00 (Reference)		1.00 (Reference)	
T2	1.20 (0.87–1.66)	0.259	1.23 (0.89–1.71)	0.215	1.14 (0.81–1.60)	0.441
T3	1.75 (1.20–2.56)	0.004	2.17 (1.45–3.26)	<.001	1.79 (1.16–2.76)	0.009
*P* for trend	1.32 (1.09–1.60)	0.005	1.49 (1.22–1.82)	<.001	1.34 (1.08–1.65)	0.006
365d						
T1	1.00 (Reference)		1.00 (Reference)		1.00 (Reference)	
T2	1.14 (0.87–1.51)	0.342	1.10 (0.83–1.47)	0.494	1.00 (0.75–1.34)	1
T3	1.62 (1.16–2.27)	0.004	1.88 (1.32–2.68)	<.001	1.49 (1.02–2.16)	0.038
*P* for trend	1.27 (1.07–1.50)	0.006	1.40 (1.18–1.68)	<.001	1.25 (1.04–1.51)	0.015

**Figure 4:**
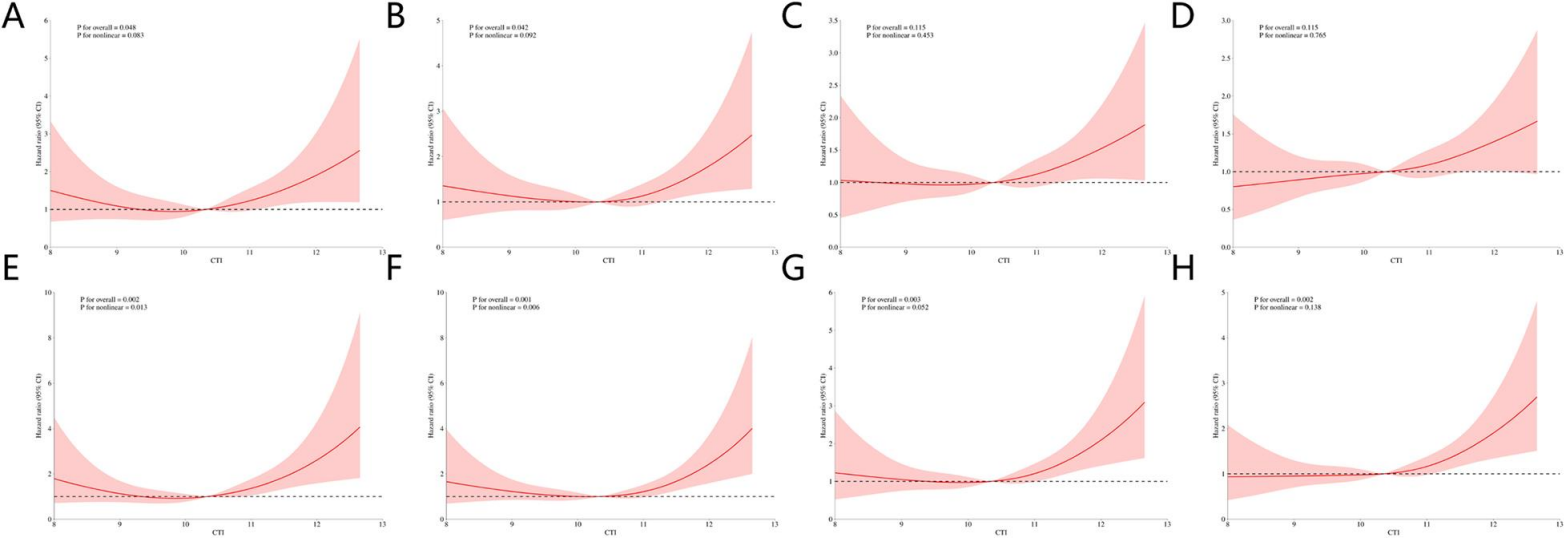
RCS regression analysis. **(A–D)** RCS regression analysis of the association of the CTI with all-cause mortality at 30-day, 90-day, 180-day, and 365-day without adjusting for covariates. **(E–H)** RCS regression analysis of the association of the CTI with all-cause mortality at 30-day, 90-day, 180-day, and 365-day after adjusting for covariates.

RCS regression curves were generated to examine the association between CTI and all-cause mortality at multiple follow-up time points (Figure 4). Across all follow-up time points, similar patterns were observed in the association between CTI and mortality, with inflection points consistently occurring at approximately 10.34. For 30-day mortality, the unadjusted model showed an overall association that approached statistical significance (*P* for overall = 0.048), whereas the test for nonlinearity was not significant (*P* for nonlinear = 0.083). The curve suggested an approximately U-shaped association, with the HR initially decreasing as CTI increased, reaching its lowest point at a CTI of approximately 10.34, and increasing thereafter. After multivariable adjustment, both the overall association and the nonlinear association became significant (*P* for overall = 0.002; *P* for nonlinear = 0.013), and the curve appeared more stable. Similar trends were observed for 90-, 180-, and 365-day mortality, indicating a consistent nonlinear association between the CTI and mortality across both short- and long-term follow-up periods, independent of other clinical confounders.

### 3.4 Subgroup analysis

**Figure 5:**
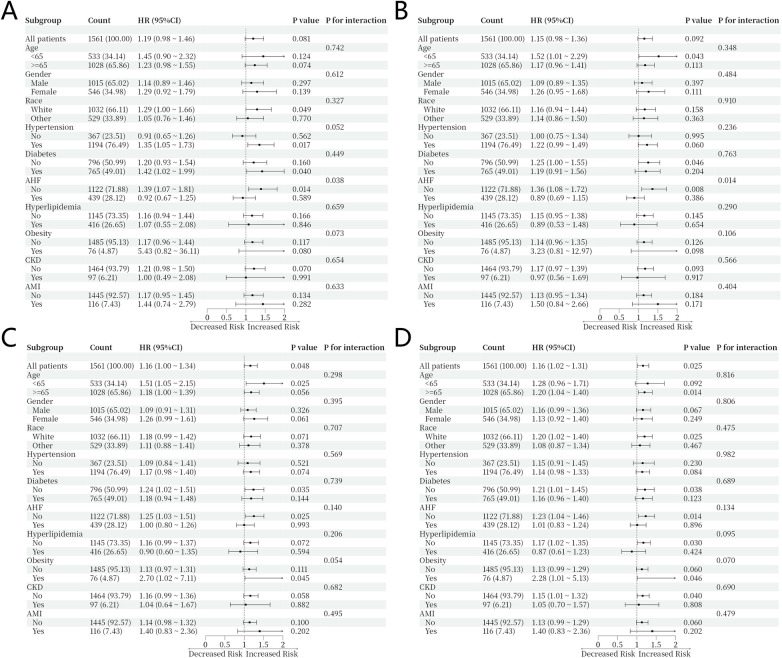
Forest plot of subgroup analyses. Forest plots of the subgroup analyses of the relationship between all-cause mortality and CTI in patients at 30-day **(A)**, 90-day **(B)**, 180-day **(C)**, and 365-day **(D).**

The consistency of the association between CTI and all-cause mortality across clinical subgroups was assessed in patients stratified by age, sex, race, hyperlipidemia, diabetes, acute heart failure, hypertension, obesity, chronic kidney disease, and acute myocardial infarction. The results are presented in forest plots (Figure 5). In most subgroups, higher CTI levels were consistently associated with increased all-cause mortality, indicating that the prognostic value of CTI was broadly applicable across patients with different clinical characteristics. No significant interactions were detected between CTI and most of the subgroups (all *P* > 0.05). An exception was observed for acute heart failure, in which modest interactions with CTI were observed at 30- and 90-day (*P* for interaction = 0.038 and 0.014), suggesting that the strength of the association may differ in this subgroup.

### 3.5 Mediation analysis

To explore potential pathways linking CTI to mortality, we conducted a bootstrap-based mediation analysis using APSIII, OASIS, LODS, SOFA, and GCS as candidate illness-severity mediators of the association between CTI and mortality (Supplementary Table S6). The results showed that APSIII and LODS exhibited relatively stable partial mediating effects. The mediation proportions for APSIII were 19.12%, 17.29%, 16.39%, and 17.59% for 30-, 90-, 180-, and 365-day mortality, respectively, whereas those for LODS were 11.13%, 10.06%, 9.54%, and 10.08%, respectively. These findings suggest that the association between CTI and mortality risk may be partially mediated by overall illness severity. In contrast, the mediating effects of the other severity scores were relatively weak. However, because both CTI and the severity scores were derived from information collected during the early ICU admission window, whereas deaths occurred during hospitalization or follow-up, these mediation results should be interpreted as exploratory. They support, but do not establish, a potential pathway through which CTI may influence mortality risk by reflecting or aggravating overall illness severity.

### 3.6 Sensitivity analysis

Three sensitivity analyses were conducted. First, when CTI was analyzed as a continuous variable in multivariable Cox proportional hazards models, the results remained consistent with those of the categorical analyses, with no substantive changes (Supplementary Table S7). Second, E-values were calculated based on fully adjusted Model 3, with E-values of 3.71, 3.09, 2.98, and 2.34 at 30-, 90-, 180-, and 365-day, respectively. These relatively large E-values indicate the robustness of the findings, suggesting that an unmeasured confounder would need to be strongly associated with both exposure and outcome (HR ≥2.34) to fully explain the observed association between CTI and mortality. Finally, when logistic regression models were used as alternatives to Cox models, the results remained consistent and stable (Supplementary Table S8). Collectively, these sensitivity analyses support the robustness of the association between CTI and all-cause mortality in this study.

### 3.7 Performance of CTI in predicting the mortality risk

#### 3.7.1 Comparison of the predictive performance between CTI and individual indicators

**Figure 6:**
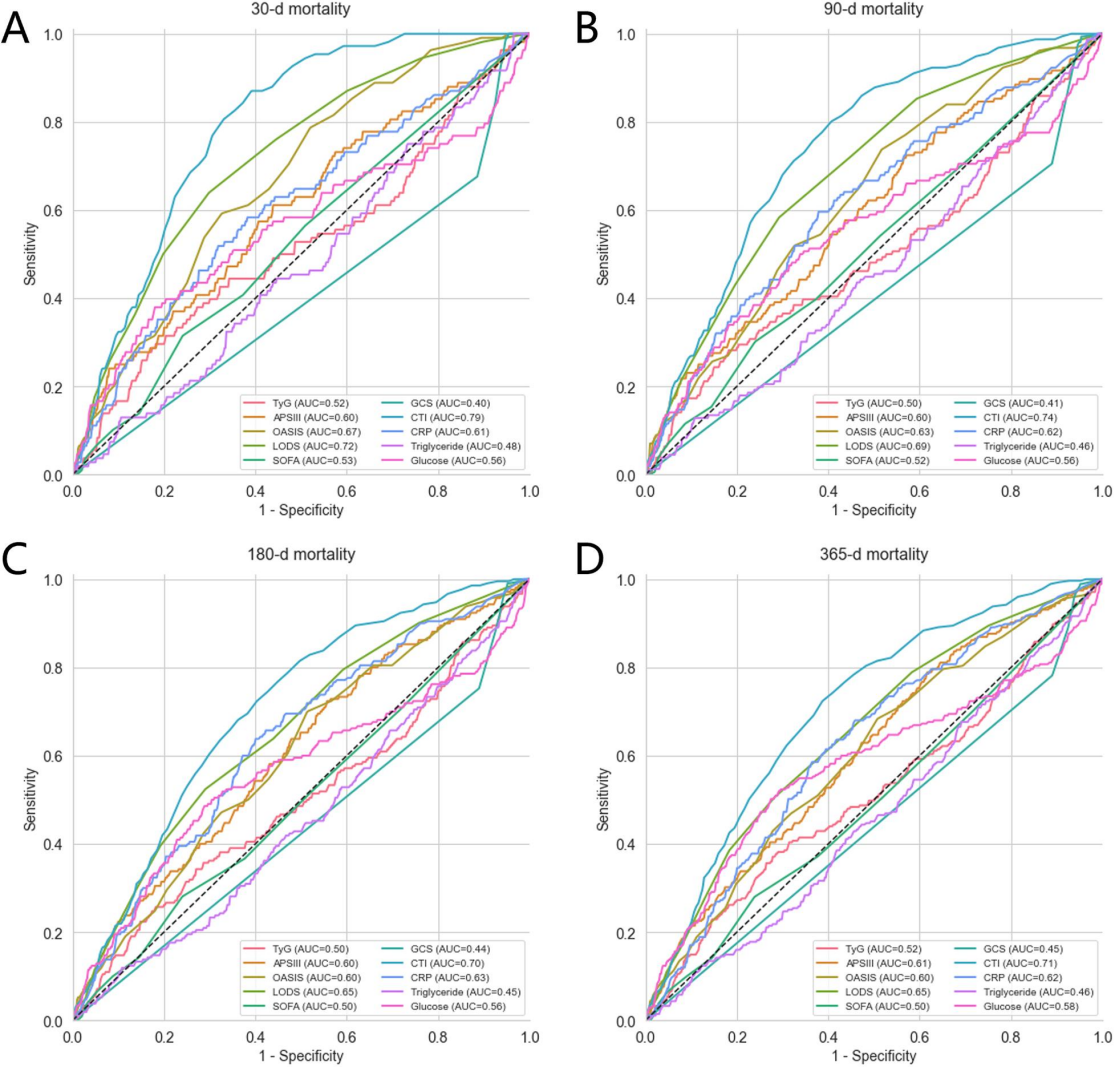
ROC curves for individual predictors. **(A)** ROC curves for predicting 30-day all-cause mortality. **(B)** ROC curves for predicting 90-day all-cause mortality. **(C)** ROC curves for predicting 180-day all-cause mortality. **(D)** ROC curves for predicting 365-day all-cause mortality.

ROC curves were used to compare the predictive performance of CTI with that of nine other indicators (TyG, APSIII, OASIS, LODS, SOFA, GCS, CRP, triglyceride, and glucose) for 30-, 90-, 180-, and 365-day all-cause mortality in critically ill patients with CAD (Figure 6). CTI consistently outperformed the other indices in predicting mortality across all time points, with AUC values of 0.79 for 30-day mortality, 0.74 for 90-day mortality, 0.70 for 180-day mortality, and 0.71 for 365-day mortality, indicating favorable predictive performance. Moreover, DeLong tests showed that the AUCs of CTI were significantly higher than those of each comparator index at all time points, with all comparisons yielding *P* values < 0.05. Detailed ROC results are presented in Supplementary Table S9, and the corresponding DeLong test results are provided in Supplementary Table S10. Longitudinal comparisons revealed that the AUCs of CTI gradually decreased over time, suggesting that CTI had greater predictive value for short-term mortality than for long-term mortality.

#### 3.7.2 Development and validation of machine learning models

Six ML models were constructed and validated in this study to predict all-cause mortality in critically ill patients with CAD at different time points. The incremental predictive value of CTI was systematically evaluated, and model interpretability and clinical applicability were enhanced through SHAP analysis and the development of a web-based application.

**Figure 7:**
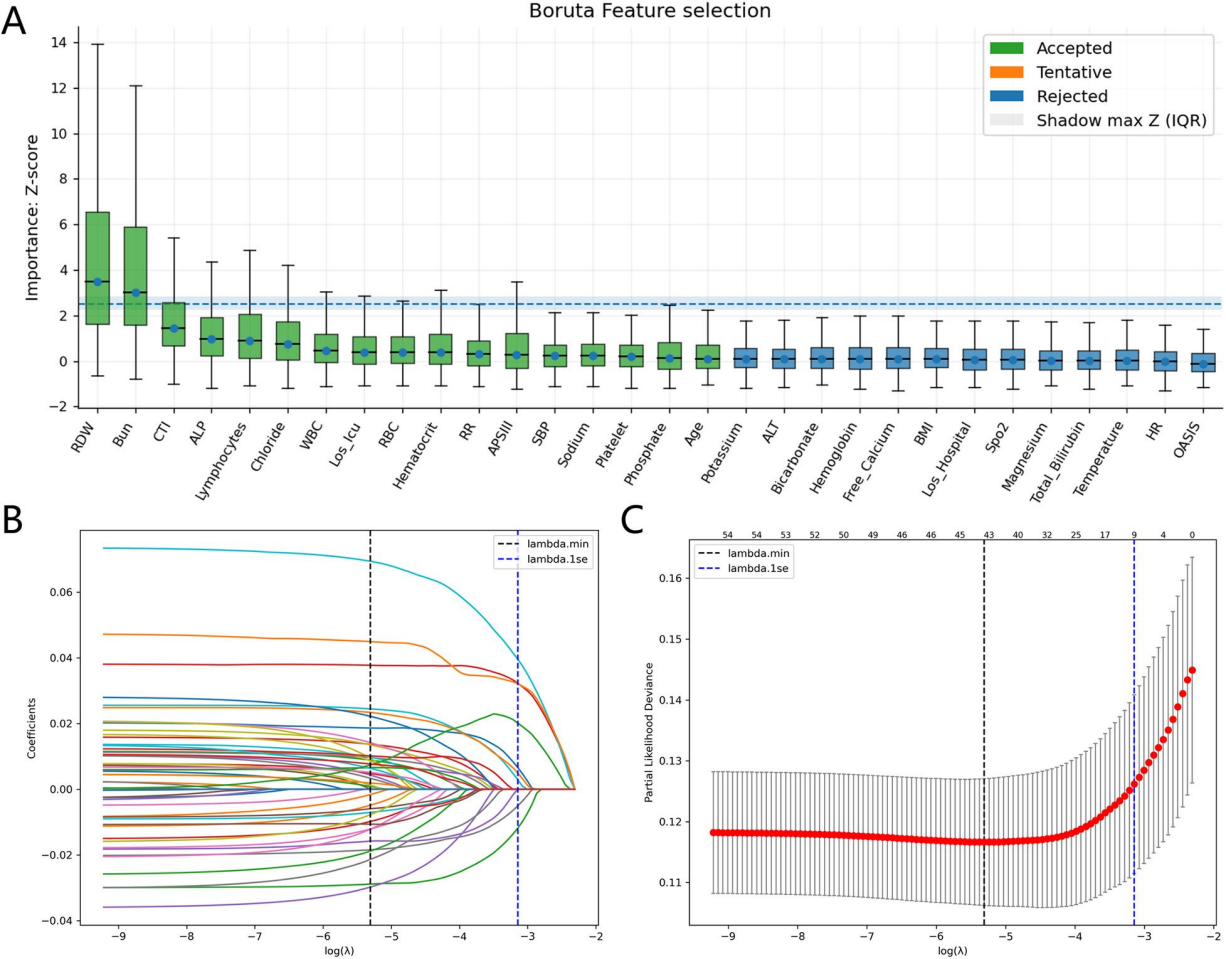
Feature selection. **(A)** Feature selection based on the Boruta algorithm. **(B)** LASSO coefficient profiles. **(C)** Selection of the tuning parameter (*λ*) in the LASSO model via 10-fold cross-validation based on the minimum criteria.

The analytic cohort from the MIMIC-IV database was randomly divided into a training set (*N* = 1,093) and an internal validation set (*N* = 468) at a 7:3 ratio. The Boruta algorithm and LASSO regression were employed for feature selection in the training set (Figure 7). The intersection of features selected by these two methods (Supplementary Table S11) and variables that were significant in univariate Cox analysis was used to identify 15 variables for model construction, including 14 baseline clinical features and CTI.

SMOTE was applied to address the class imbalance between positive and negative samples in the training set. The selected features were divided into two sets: a baseline set containing only clinical features and a combined set that additionally included CTI. Based on these two feature sets, six ML models—LR, KNN, SVC, RF, XGBoost, and CNN—were developed to predict mortality in critically ill patients with CAD. The optimal hyperparameters for each ML model were determined using five-fold cross-validation and grid search, and are presented in Supplementary Table S12.

**Figure 8:**
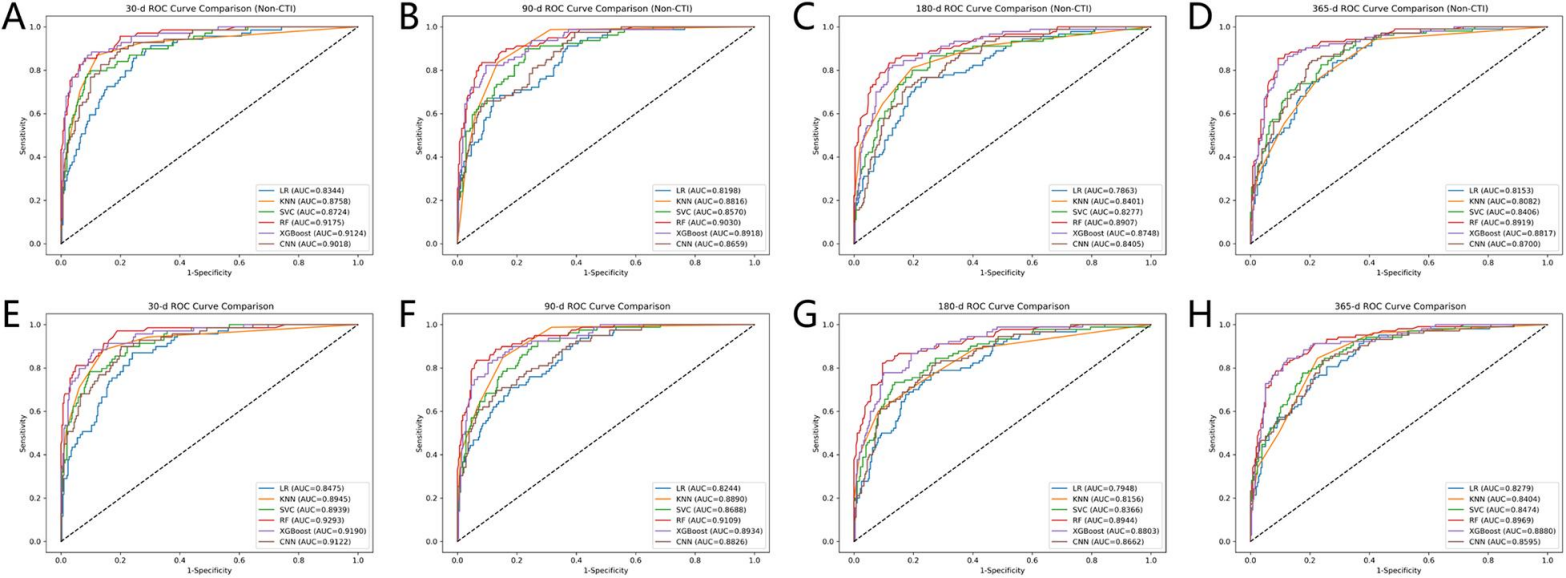
Comparison of the ROC curves from the six machine learning algorithms. ROC curves for predicting 30-day **(A)**, 90-day **(B)**, 180-day **(C)**, and 365-day **(D)** all-cause mortality without the inclusion of the CTI. ROC curves for predicting 30-day **(E)**, 90-day **(F)**, 180-day **(G)**, and 365-day **(H)** all-cause mortality with the inclusion of the CTI.

**Figure 9:**
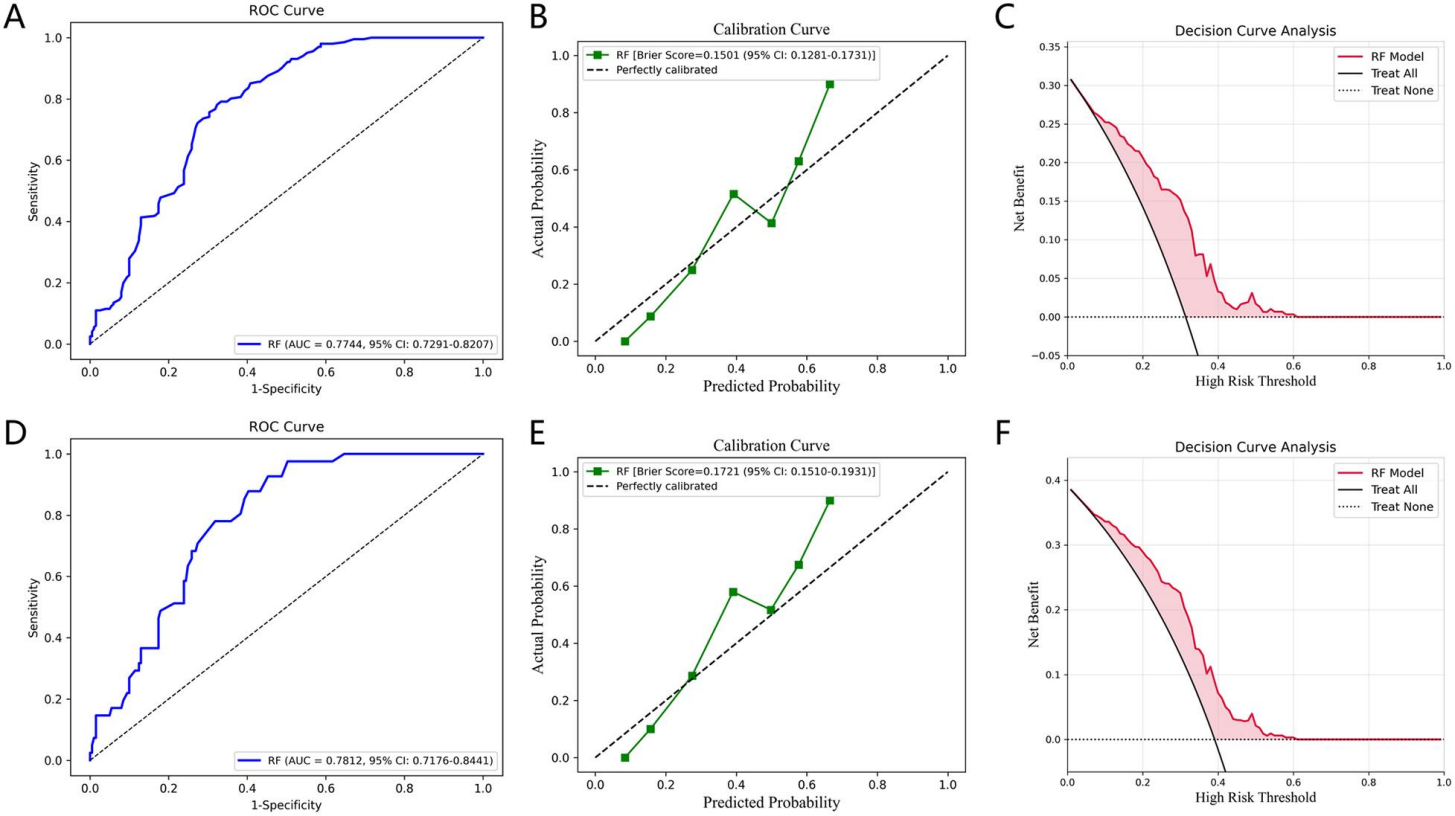
Predictive performance of the RF model in the external validation cohort. **(A)** ROC curve for the eICU-CRD cohort. **(B)** Calibration curve for the eICU-CRD cohort. **(C)** Decision curve analysis of the eICU-CRD cohort. **(D)** ROC curve for the SRHIP cohort. **(E)** Calibration curve for the SRHIP cohort. **(F)** Decision curve analysis of the SRHIP cohort.

In the internal test set, model performance was comprehensively evaluated by plotting ROC curves (Figure 8) and comparing multiple metrics, including AUC, F1-score, accuracy, precision, recall, NRI, and IDI to select the optimal model (Supplementary Table S13, S14). The optimal model was externally validated in the eICU-CRD and SRHIP cohorts for predicting 30-day all-cause mortality in patients with CAD. Its generalizability was further assessed using ROC, calibration, and DCA curves (Figure 9).

The results showed that incorporating CTI improved the predictive performance of all ML models to varying degrees across different follow-up time points, confirming its incremental predictive value. For 30-day mortality prediction (Figures 8A,E), the AUC increased from 0.8344 to 0.8475 for LR, from 0.8758 to 0.8945 for KNN, from 0.8724 to 0.8939 for SVC, from 0.9175 to 0.9293 for RF, from 0.9124 to 0.9190 for XGBoost, and from 0.9018 to 0.9122 for CNN. The greatest improvements in AUC were observed for the KNN and SVC models, with increases of 0.0187 and 0.0215, respectively, suggesting that CTI had a more pronounced effect on these algorithms. Even among models with strong baseline performance (RF, XGBoost, and CNN), CTI further improved predictive performance, with RF achieving the highest AUC (0.9293). Consistent with the AUC results, NRI and IDI analyses further showed that the incorporation of CTI improved reclassification performance in most models across different follow-up time points. This improvement was particularly consistent for 90-day mortality, for which all models showed positive NRI and IDI values. For 30-day mortality, positive reclassification gains were also observed in several models, including LR (NRI = 0.2630, IDI = 0.0042), SVC (NRI = 0.2728, IDI = 0.0059), and RF (NRI = 0.0493, IDI = 0.0005). Across algorithms, LR, SVC, XGBoost, and RF demonstrated relatively stable positive reclassification gains, whereas the incremental value of CTI was less consistent for KNN and CNN at certain follow-up time points. However, the predictive performance declined with longer follow-up, indicating that CTI contributed more to predicting short-term mortality than to long-term mortality, which is consistent with previous findings. A representative patient example illustrating the absolute change in predicted mortality risk after the incorporation of CTI is provided in the “A representative patient example” section of the Supplementary Material.

For external validation, the best-performing RF model identified in the internal test cohort was first evaluated in the eICU-CRD cohort, which included 242 patients and 41 death events. The model showed good generalizability, with an AUC of 0.7744 (95% CI: 0.7291–0.8207) and a Brier score of 0.1501 (95% CI: 0.1281–0.1731). DCA also indicated a clear net clinical benefit across a range of threshold probabilities. To further evaluate the model's geographic robustness, cross-region validation was conducted using the SRHIP cohort, which included 105 patients and 21 death events. In this independent real-world cohort, the model achieved a comparable performance with an AUC of 0.7812 (95% CI: 0.7176–0.8441) and a Brier score of 0.1721 (95% CI: 0.1510–0.1937). It also showed favorable decision curve patterns, supporting its applicability across distinct populations and healthcare settings.

**Figure 10:**
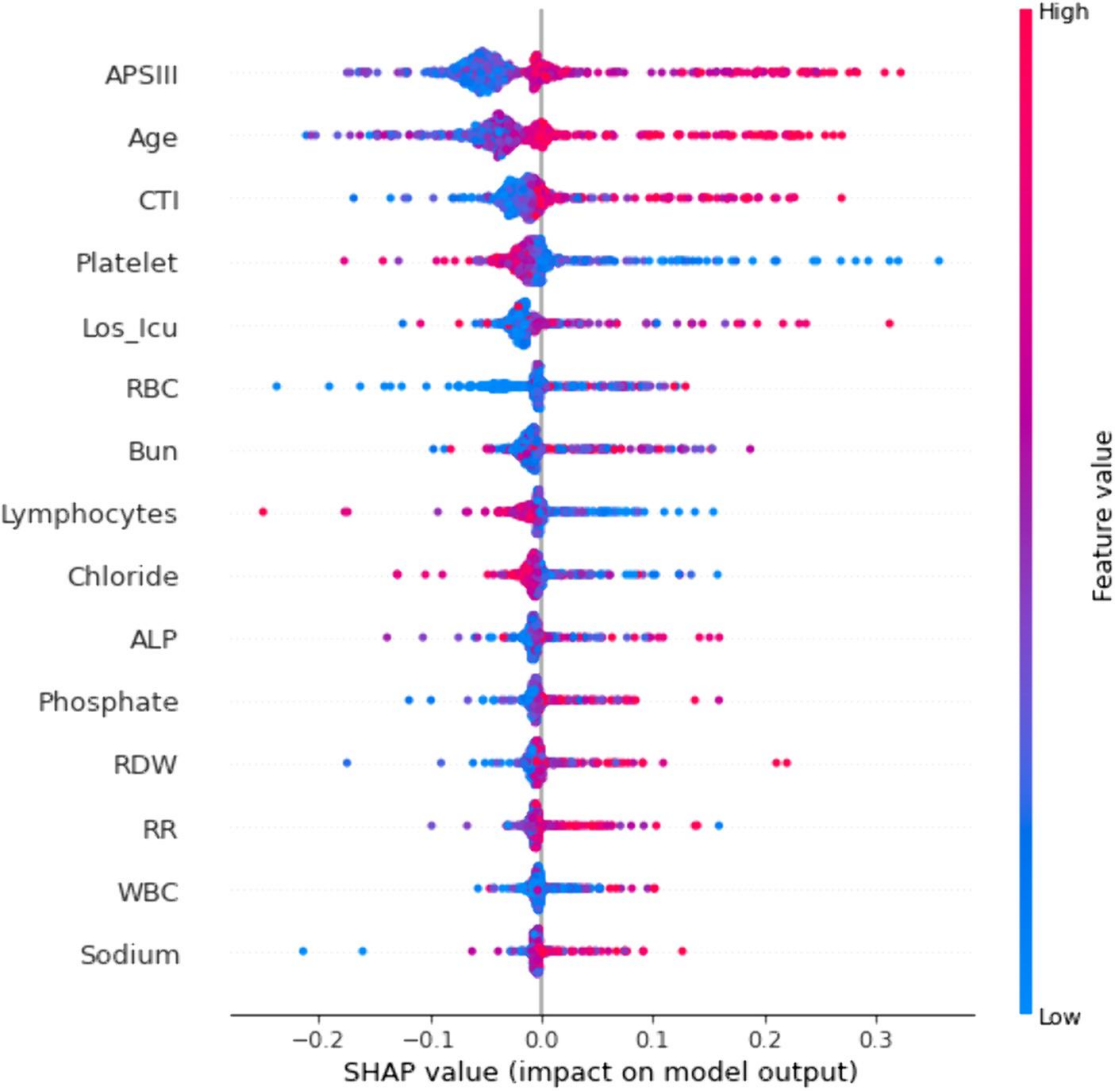
SHAP interpretability beeswarm plot.

**Figure 11:**
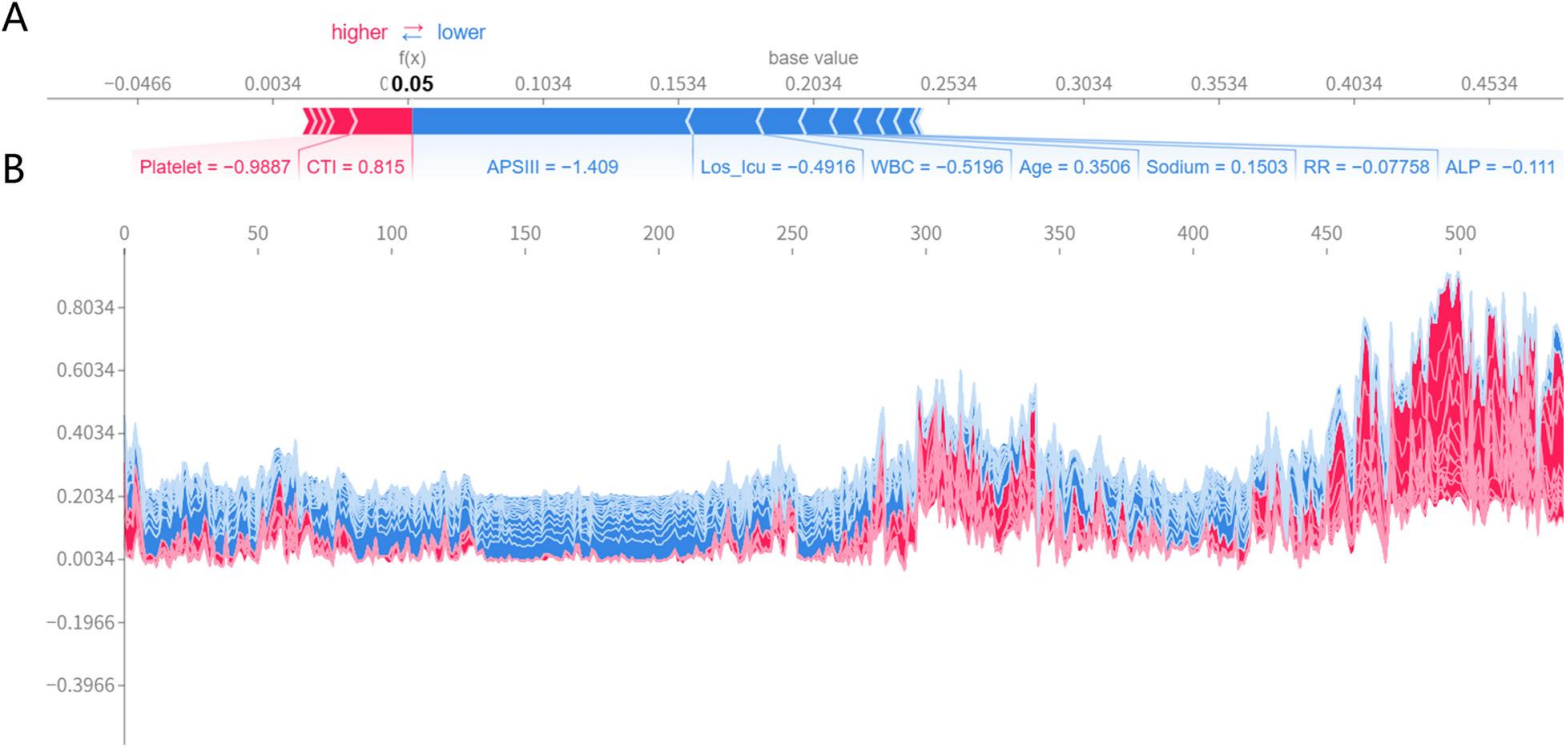
Global and local interpretability analysis of SHAP. **(A)** SHAP force plot for an individual patient. **(B)** SHAP force plot for all patients.

SHAP method was applied to calculate the contributions of features to the optimal model and to make the ML model more intuitive and understandable (Figure 10). Global and local visualizations were further generated (Figure 11) to illustrate the importance of the variables in the prediction process.

As shown in Figure 10, APSIII, age, and CTI were the most important contributors to mortality risk, and all were risk-promoting factors, consistent with clinical expectations. Figure 11A illustrates an individual SHAP explanation for a patient with a low predicted mortality risk (5%). Although a low platelet count and elevated CTI increased mortality risk to some extent, the overall risk remained low because of the mild disease severity (low APSIII score) and other protective factors. These findings suggest that platelet count and CTI should be closely monitored during clinical management, while other favorable conditions should also be maintained to support a better prognosis. Figure 11B shows local explanation plots for all patients in the test set, ordered by similarity in predicted values and displayed after a 90° counterclockwise rotation. These findings demonstrate that the model can provide individualized SHAP explanations for each patient, highlighting its interpretability and potential clinical applicability.

**Figure 12:**
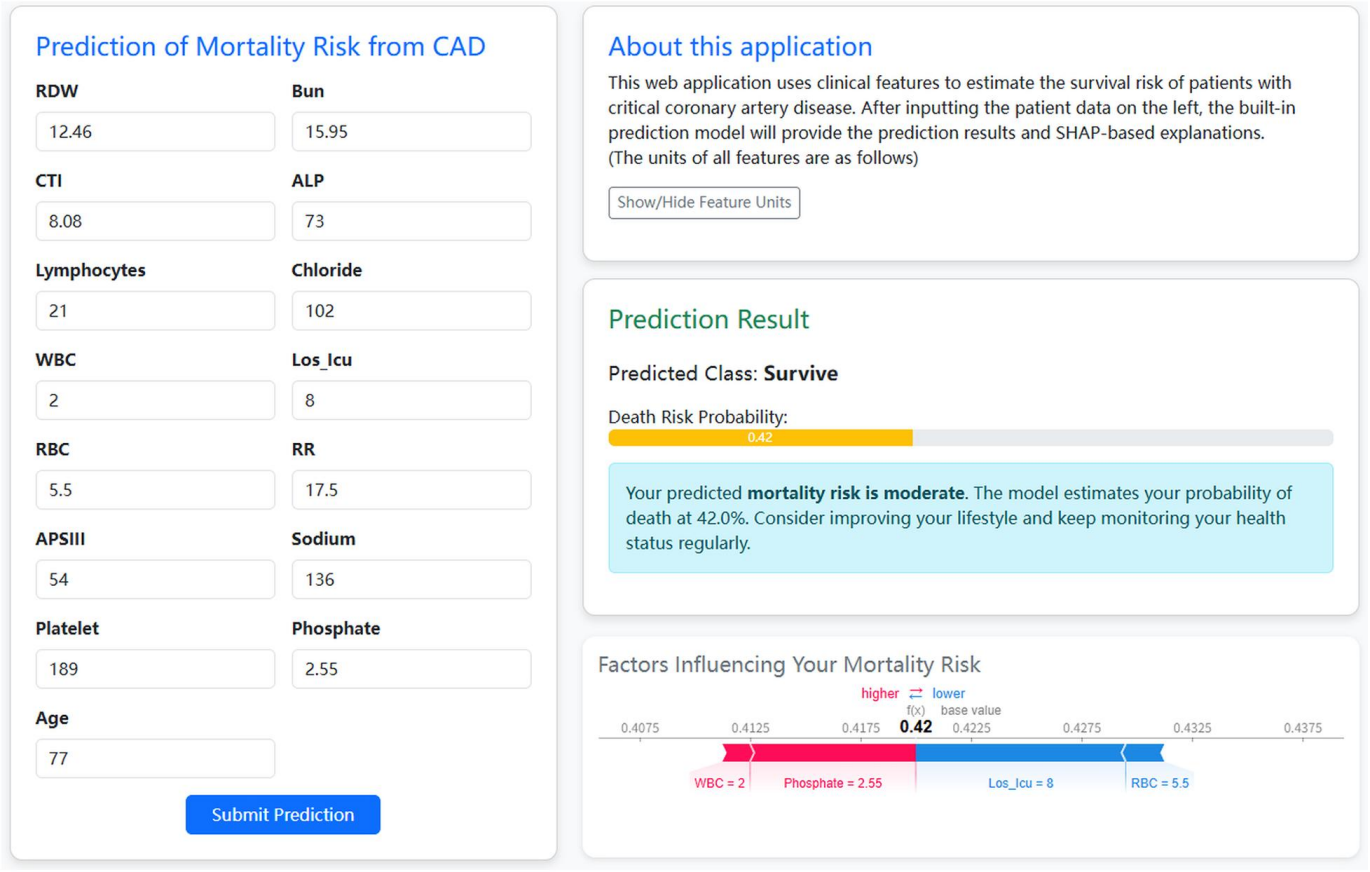
Example of web application usage.

Finally, a web-based application was developed to enhance the clinical applicability of the ML model (Figure 12). The application was developed using the Python Flask framework and deployed on Tencent Cloud Lighthouse (URL: http://62.234.14.22:5002/). The main interface incorporated 15 selected key variables. After patient-specific information was entered, the program automatically calculated mortality risk and generated SHAP visualizations. The application incorporated the optimal binary classification ML model. For each patient, the model outputs a predicted probability ranging from 0 to 1. By default, a threshold of 0.50 is used to convert predicted probability into a binary label: patients with a probability < 0.50 are classified as “Survive,” whereas those with a probability ≥0.50 are classified as “Non-survive.” However, in clinical practice, the predicted probability should be interpreted only as reference prognostic information, and treatment decisions should be made on the basis of the patient's overall clinical condition and the clinician's judgment.

## 4 Discussion

This study is the first to systematically evaluate the associations between CTI and short- and long-term mortality in critically ill patients with CAD in a multicenter cohort. The results demonstrated that elevated CTI levels were significantly associated with increased short- and long-term all-cause mortality and a prolonged hospital stay, with greater accuracy in predicting short-term mortality. In multivariable Cox proportional hazards models, these associations remained robust after adjustment for potential confounders. The RCS regression analysis revealed significant nonlinear U-shaped relationships between CTI and both short- and long-term mortality that were independent of other clinical covariates. Subgroup analyses revealed no significant interactions with long-term all-cause mortality, whereas a significant interaction was observed in the acute heart failure subgroup with short-term mortality. Mediation and sensitivity analyses further supported the robustness of the findings. Moreover, compared with existing disease severity scores or individual predictive indicators, CTI demonstrated superior predictive performance. Incorporating CTI into ML models significantly improved predictive performance, and SHAP-based feature importance analysis identified CTI as one of the key contributing variables.

Compared with single indicators, CTI, as a dual-pathway biomarker integrating inflammation and IR, provides broader pathological coverage and superior performance for predicting the cardiovascular risk. It is derived from the TyG index, a biomarker of IR, and CRP levels, a well-established marker of inflammation. Extensive evidence indicates that the TyG index has robust prognostic value for assessing disease severity and predicting the clinical outcomes in patients with CAD. For example, SM et al. (32)found that an elevated TyG index was independently associated with an increased CAD risk and adverse cardiovascular outcomes. In asymptomatic populations without traditional cardiovascular risk factors, the TyG index has also been identified as an independent predictor of the subclinical CAD (33). Similarly, Wan et al. (34) using data from the Shanghai Suburban Adult Cohort and Biobank, observed a positive association between an elevated TyG index and CAD incidence, suggesting its utility in identifying high-risk individuals at an early stage. In addition, several TyG-derived indices, such as TyG-WHtR, TyG-WC, TyG-BMI, and TyG-ABSI, have demonstrated significant clinical value in predicting prognosis across various diseases (35, 36). Meanwhile, CRP, a key marker of inflammation, is also an important risk factor for CAD. Luo et al. (37) reported that elevated baseline CRP levels were independent predictors of major adverse cardiovascular events (MACEs), cardiovascular mortality, and all-cause mortality in CAD patients, providing important prognostic information for patients with stable CAD. A comprehensive study based on the UK Biobank further showed a significant association between CRP levels and the risk of CAD recurrence (38). Song et al. (39) also emphasized that CRP is not only an independent risk factor for CAD onset but also a strong predictor of disease progression and severity.

Compared with the TyG index or CRP alone, the composite CTI offers several advantages. First, CTI simultaneously captures two key pathological mechanisms, chronic low-grade inflammation and IR. This reduces the bias associated with reliance on a single biomarker and enables a more comprehensive assessment of atherosclerotic progression, consistent with the findings of Cui et al. (40). Second, CTI has shown better predictive performance than single indicators in multiple studies. For example, a study based on NHANES data showed that the CTI was significantly associated with both the CVD incidence and mortality, establishing it as a distinct predictor of cardiovascular risk (41). In another analysis of stroke risk stratification, CTI consistently outperformed CRP and the TyG index when each was used alone (42). Third, the CTI relies solely on routine laboratory measures (CRP, triglyceride, and fasting glucose), making it inexpensive, readily available, and suitable for physical examinations, primary care, and large-scale screening. Finally, CTI may also be valuable for longitudinal monitoring, as cumulative exposure and trajectory changes have been independently associated with incident CVD risk. Individuals with persistently high or increasing CTI levels have a significantly higher risk of events, suggesting that incorporating CTI into follow-up management and dynamic assessment may provide information beyond that offered by single indicators (43).

Although the exact pathophysiological mechanisms linking CTI to adverse outcomes in patients with CAD remain incompletely elucidated, CTI reflects both IR and systemic inflammation, and its association with clinical outcomes is likely mediated by multiple overlapping pathways. First, IR directly impairs endothelial function, characterized by impaired PI3K–Akt–eNOS signaling and excessive MAPK pathway activation, leading to endothelial dysfunction and a proinflammatory state (44). Second, CRP acts not only as a marker of inflammation but also as an active participant in atherosclerosis. It induces endothelial activation, upregulates adhesion molecules and tissue factor expression, promotes inflammation and complement activation, and reduces fibrous cap stability, thereby increasing thrombotic risk (45). Moreover, hypertriglyceridemia and hyperglycemia exacerbate oxidative stress and injure the endothelial glycocalyx through lipotoxic and glucotoxic effects (46). Abnormal activation of the renin–angiotensin–aldosterone system (RAAS) further increases the hemodynamic load and the risk of myocardial ischemia (47). Platelet hyperreactivity also contributes to a prothrombotic state through interactions with monocytes to upregulate tissue factor (TF) expression and synthesis, further increasing the thrombotic risk (48). Collectively, these mechanisms support CTI as a composite indicator of metabolic dysregulation and inflammatory burden, which may explain its strong association with adverse outcomes in patients with CAD.

The observed U-shaped association with mortality suggests that elevated CTI reflects worsening inflammation and metabolic dysregulation, whereas abnormally low CTI may indicate an adverse clinical state. Specifically, in the ICU setting, low CTI values may reflect impaired physiological reserve and an inadequate inflammatory or metabolic response to severe stress. Clinically, this pattern may be observed in patients with malnutrition or cachexia, chronic energy depletion, advanced hepatic dysfunction accompanied by impaired CRP synthesis and disordered lipid and glucose metabolism, or severe immunosuppression and immune paralysis. Under such conditions, inflammatory and metabolic markers may remain low not because the patient is less severely ill, but because the host response is blunted or exhausted. In addition, reverse causation cannot be excluded. Severe critical illness itself may suppress inflammatory and metabolic markers during the early phase of ICU admission, such that a low CTI may partly represent an epiphenomenon of advanced disease severity rather than a truly protective metabolic profile. Therefore, the excess risk observed at the lower end of the CTI spectrum may identify a subgroup of patients characterized by frailty, poor nutritional status, organ dysfunction, or an inadequate stress response, all of which may contribute to a worse prognosis (49, 50).

In addition, subgroup analyses revealed a significant interaction between CTI and acute heart failure with respect to short-term mortality. Specifically, the prognostic effect of CTI was more pronounced in patients without acute heart failure, whereas no significant association was observed in those with acute heart failure. This finding may be explained by the fact that, in patients with acute heart failure, short-term outcomes are more likely to be driven by severe hemodynamic instability, congestion, neurohormonal activation, and multi-organ dysfunction, thereby reducing the relative incremental prognostic value of CTI (51). In contrast, among patients without acute heart failure, CTI may more directly reflect the inflammatory and metabolic burden associated with CAD and therefore show a stronger association with mortality. Meanwhile, the direction of the association between CTI and mortality was generally consistent between patients with AMI and those without AMI, although no significant interaction was observed. This suggests that the prognostic relevance of CTI may not be restricted to a specific AMI phenotype; however, these findings should be interpreted cautiously given the limited sample size of the AMI subgroup.

Accurate prognostic assessment remains challenging in the complex ICU setting. Electronic medical record systems, nomograms, and ML approaches have been widely used for disease prediction and are increasingly recognized as useful tools for evaluating clinical predictive performance (52). Previous studies have shown that ML can achieve good performance in predicting mortality risk in patients with CAD. For example, studies in patients with acute coronary syndrome (ACS) and acute myocardial infarction (AMI) have shown that ML such as XGBoost can predict in-hospital and 1-year mortality with acceptable discrimination and calibration, including in external validation, highlighting their potential for clinical translation (53, 54). Similarly, Ye et al. (55) reported that ML could reliably predict in-hospital mortality in critically ill patients with CAD. In this study, CTI was incorporated into ML frameworks to develop prediction models for both short- and long-term outcomes in critically ill patients with CAD. Among the evaluated algorithms, RF achieved the best overall performance in both the internal test set and the external validation cohort. The addition of CTI improved model performance across all ML models, although the magnitude of improvement varied. The incremental gains were modest, likely because the baseline models already included strong predictors, such as APSIII and age, which captured a substantial proportion of mortality risk. In this context, the contribution of a single composite biomarker would be expected to be additive rather than transformative. Nevertheless, the consistent improvement observed after the inclusion of CTI suggests that this index captures prognostic information beyond conventional clinical variables, particularly an inflammatory–metabolic dimension of risk that may not be fully reflected by traditional severity scores. Accordingly, even modest improvements in model performance may be clinically meaningful for refining risk stratification in critically ill patients with CAD. SHAP analysis further showed that APSIII, age, and CTI were the three most influential predictors, consistent with clinical expectations and further supporting the prognostic value of CTI in this population. To enhance translational applicability, the optimal model was deployed as an interactive web application that enables seamless integration from data entry to real-time prediction and interpretable output. This tool may facilitate rapid and reproducible individualized risk assessment and support integration into clinical decision support systems. It can also provide a practical foundation for ongoing external validation and iterative model refinement, thereby promoting the application of precision medicine in CAD risk management.

In summary, this exploratory study helped clarify the predictive validity of CTI across different follow-up periods, supporting its discriminative ability and potential clinical applicability. However, several limitations should be acknowledged. First, although this study included data from a U.S. database and an independent real-world cohort from Shenzhen, China, the external validation cohorts, particularly the SRHIP cohort, were relatively small compared with the training cohort. Moreover, the noticeable decline in model performance from internal to external validation may indicate optimism in the model development process. Therefore, performance estimates derived from the external validation cohorts, including calibration curve and DCA, may be less precise. Second, the study used all-cause mortality as the primary outcome and did not include other CAD-related endpoints, such as cardiac death, reinfarction, or stroke, resulting in an incomplete characterization of disease-specific risk. In addition, a validated CAD-specific risk score, such as GRACE or TIMI, could not be incorporated for direct comparison with CTI because of data limitations, which may have limited the disease-specific contextualization of its prognostic performance. More detailed stratification of CAD phenotypes, such as ACS, stable CAD, and cardiogenic shock, was not fully available in the present study, which may also have limited a more nuanced evaluation of phenotype-specific prognostic differences. Third, although rigorous covariate adjustment and E-value analysis were performed, residual confounding could not be excluded because of the retrospective study design, and causality could not be inferred. Fourth, only baseline CTI was assessed, and dynamic changes during hospitalization or follow-up were not captured. In addition, because blood glucose measurements obtained in the ICU are usually not performed under strict fasting conditions, these values may not fully reflect the patients’ underlying metabolic status. This may have introduced non-differential measurement error in CTI estimation, potentially attenuating the true associations with mortality. Future studies should incorporate diverse cardiovascular endpoints and disease phenotypes in multicenter prospective cohorts, integrate dynamic CTI trajectories with treatment information, and apply competing-risk models and causal-inference frameworks to clarify the underlying mechanisms. External validation and recalibration across multiple centers are also needed to assess the real-world utility of CTI in different healthcare settings.

## 5 Conclusions

This study showed for the first time the clinical value of CTI in predicting the short- and long-term mortality risk in critically ill patients with CAD. CTI was independently associated with all-cause mortality, showed greater predictive value for short-term outcomes, and outperformed traditional scoring systems. The nonlinear U-shaped association indicated that both low and high CTI levels were associated with increased risk. Incorporating CTI into ML models further improved predictive performance, highlighting its potential as a novel composite biomarker. Routine monitoring of CTI may facilitate risk stratification and prognostic management in critically ill patients with CAD, enabling the early identification of high-risk individuals and timely intervention to improve outcomes.

## Data Availability

Publicly available datasets were analyzed in this study. This data can be found here: https://physionet.org/content/mimiciv/3.1/; https://physionet.org/content/eicu-crd/2.0/. The SRHIP dataset contains real-world inpatient records from Shenzhen and is not publicly available due to institutional and ethical restrictions. Access to the SRHIP data may be granted upon reasonable request to the corresponding authors and approval by the relevant institutional ethics committee.
