# Supplementary material for: Development and validation of the C-reactive protein–triglyceride-glucose index for predicting short- and long-term mortality in critically ill patients with coronary artery disease: a multicenter cohort study
Source: Front Cardiovasc Med. 2026 May 13;13:1763569. doi: 10.3389/fcvm.2026.1763569 (PMC13216771; doi:10.3389/fcvm.2026.1763569)
Supplement: Supplementary file 1 [file Datasheet1.docx]

Supplementary Material

# Supplementary Tables

Supplementary Table S1 Baseline characteristics of patients from the MIMIC-IV database.

| **Variables** | **Total (n = 1561)** | **T1 (n = 649)** | **T2 (n = 698)** | **T3 (n = 214)** | **P value** |
| --- | --- | --- | --- | --- | --- |
| **Demographics** |  |  |  |  |  |
| Age (years) | 70.19 (61.74, 77.82) | 69.83 (60.43, 76.87) | 70.63 (61.75, 77.71) | 71.44 (63.12, 78.45) | 0.015 |
| BMI (kg/m2) | 29.73 (27.34, 32.59) | 29.15 (26.71,31.50) | 29.92 (27.93,32.93) | 30.76 (28.61,34.98) | <.001 |
| Gender, n(%) |  |  |  |  | 0.234 |
| Male | 1015 (65.02) | 424 (65.33) | 442 (63.32) | 149 (69.63) |  |
| Female | 546 (34.98) | 225 (34.67) | 256 (36.68) | 65 (30.37) |  |
| Marriage, n(%) |  |  |  |  | 0.113 |
| Married | 876 (56.12) | 385 (59.32) | 370 (53.01) | 121 (56.54) |  |
| Single | 390 (24.98) | 153 (23.57) | 179 (25.64) | 58 (27.10) |  |
| Other | 295 (18.90) | 111 (17.10) | 149 (21.35) | 35 (16.36) |  |
| Race, n(%) |  |  |  |  | 0.264 |
| White | 1032 (66.11) | 435 (67.03) | 466 (66.76) | 131 (61.21) |  |
| Other | 529 (33.89) | 214 (32.97) | 232 (33.24) | 83 (38.79) |  |
| **Vital Signs** |  |  |  |  |  |
| HR (bpm) | 81.40 (74.28, 90.47) | 80.70 (73.26,89.27) | 81.99 (74.73,90.15) | 83.55 (75.27,93.45) | 0.007 |
| SBP (mmhg) | 113.08 (106.43, 121.27) | 112.86 (106.46,120.67) | 113.35 (106.44,121.92) | 113.29 (106.23,121.54) | 0.749 |
| DBP (mmhg) | 59.21 (54.00, 66.20) | 59.07 (54.48,65.71) | 58.84 (53.63,65.83) | 61.27 (53.70,68.33) | 0.223 |
| MBP (mmhg) | 75.20 (70.70, 81.23) | 75.39 (71.09,80.55) | 74.75 (70.26,81.22) | 75.75 (71.37,81.99) | 0.321 |
| RR (insp/min) | 18.42 (16.70, 20.63) | 18.16 (16.44,20.12) | 18.51 (16.76,20.77) | 19.66 (17.36,22.09) | <.001 |
| Temperature (℃) | 36.75 (36.57, 36.96) | 36.72 (36.56,36.91) | 36.76 (36.56,36.96) | 36.87 (36.70,37.16) | <.001 |
| Spo2 (%) | 97.33 (96.13, 98.48) | 97.41 (96.29,98.52) | 97.37 (96.15,98.55) | 96.95 (95.78,98.15) | 0.002 |
| **Laboratory Indicators** |  |  |  |  |  |
| CRP (mg/L) | 13.90 (3.10, 80.70) | 2.70 (1.00,6.10) | 45.20 (12.62,97.55) | 150.20 (84.28,209.00) | <.001 |
| Hematocrit (%) | 32.39 (29.33, 35.85) | 33.60 (30.66,37.21) | 31.79 (28.80,34.90) | 30.61 (28.00,33.84) | <.001 |
| Platelet (K/μL) | 225.63 (183.13, 274.26) | 217.36 (181.52,261.30) | 229.39 (184.50,279.67) | 243.17 (189.33,290.19) | <.001 |
| Bun (mg/dL) | 23.07 (17.13, 36.19) | 20.60 (15.90,28.09) | 24.65 (17.78,38.60) | 34.18 (21.15,48.71) | <.001 |
| Creatinine (mg/dL) | 1.14 (0.89, 1.65) | 1.02 (0.85,1.35) | 1.18 (0.90,1.71) | 1.66 (1.09,2.58) | <.001 |
| Potassium (mEq/L) | 4.28 (4.10, 4.46) | 4.26 (4.09,4.42) | 4.29 (4.10,4.47) | 4.32 (4.10,4.51) | 0.023 |
| Sodium (g/L) | 138.44 (136.77, 139.90) | 138.67 (137.05,140.00) | 138.32 (136.75,139.86) | 137.78 (136.34,139.48) | <.001 |
| Magnesium (mg/dL) | 2.07 (1.97, 2.19) | 2.07 (1.98,2.17) | 2.07 (1.96,2.19) | 2.11 (2.00,2.23) | 0.079 |
| PT (seconds) | 13.82 (12.56, 16.78) | 13.53 (12.44,15.82) | 14.24 (12.64,17.24) | 14.09 (12.82,17.47) | <.001 |
| INR | 1.26 (1.14, 1.54) | 1.23 (1.12,1.45) | 1.28 (1.15,1.59) | 1.30 (1.17,1.59) | <.001 |
| RDW (%) | 14.72 (13.71, 16.01) | 14.30 (13.50,15.50) | 14.94 (13.84,16.27) | 15.27 (14.17,16.57) | <.001 |
| Bicarbonate (mEq/L) | 24.97 (23.52, 26.48) | 25.21 (23.89,26.63) | 25.00 (23.44,26.46) | 24.29 (22.68,25.74) | <.001 |
| eGFR (mL/min/1.73m2) | 55.78 (43.80, 65.30) | 58.12 (46.89,66.26) | 53.66 (42.34,64.35) | 56.76 (43.16,64.63) | <.001 |
| Triglyceride (mg/dl) | 141.00 (106.62, 175.00) | 121.24 (89.00,152.67) | 144.89 (116.00,177.96) | 189.32 (158.00,268.62) | <.001 |
| Fasting blood glucose (mg/dl) | 134.43 (121.21, 160.44) | 126.79 (116.60,142.50) | 136.54 (123.55,164.79) | 170.50 (140.75,212.34) | <.001 |
| WBC (K/uL) | 7.00 (2.00, 16.23) | 7.00 (2.00,14.25) | 7.00 (2.10,17.94) | 8.00 (3.00,16.94) | 0.064 |
| RBC (m/uL) | 4.45 (1.97, 10.77) | 4.00 (1.53,8.55) | 4.52 (2.00,11.62) | 7.00 (2.57,16.19) | <.001 |
| Hemoglobin (g/dL) | 9.94 (8.91, 11.21) | 10.25 (9.14,11.40) | 9.81 (8.80,11.05) | 9.86 (8.82,11.00) | <.001 |
| ALT (IU/L) | 26.00 (18.43, 45.00) | 25.33 (18.41,39.00) | 25.06 (17.60,45.70) | 31.15 (22.10,59.99) | <.001 |
| ALP (IU/L) | 90.00 (71.00, 118.43) | 83.00 (65.54,105.40) | 91.62 (72.94,123.60) | 102.87 (78.69,142.54) | <.001 |
| Total Bilirubin (mg/dL) | 0.50 (0.37, 0.72) | 0.50 (0.39,0.71) | 0.49 (0.35,0.71) | 0.51 (0.33,0.77) | 0.137 |
| Free Calcium (mmol/L) | 1.14 (1.11, 1.17) | 1.15 (1.12,1.17) | 1.14 (1.11,1.17) | 1.13 (1.10,1.15) | <.001 |
| Phosphate (mg/dL) | 3.54 (3.22, 3.98) | 3.46 (3.16,3.81) | 3.56 (3.27,4.03) | 3.80 (3.37,4.28) | <.001 |
| Chloride (mEq/L) | 104.50 (102.07, 106.65) | 105.00 (102.50,107.00) | 104.29 (102.00,106.33) | 104.27 (101.70,106.50) | 0.004 |
| Lymphocytes (%) | 17.90 (13.01, 23.31) | 19.60 (15.03,24.94) | 17.11 (12.08,22.11) | 14.98 (10.57,20.97) | <.001 |
| CTI (index) | 10.34 (9.56, 11.02) | 9.44 (9.01,9.79) | 10.73 (10.41,11.03) | 11.71 (11.53,11.94) | <.001 |
| **Comorbidities** |  |  |  |  |  |
| Hypertension, n(%) |  |  |  |  | 0.369 |
| No | 367 (23.51) | 142 (21.88) | 169 (24.21) | 56 (26.17) |  |
| Yes | 1194 (76.49) | 507 (78.12) | 529 (75.79) | 158 (73.83) |  |
| Diabetes, n(%) |  |  |  |  | <.001 |
| No | 796 (50.99) | 404 (62.25) | 321 (45.99) | 71 (33.18) |  |
| Yes | 765 (49.01) | 245 (37.75) | 377 (54.01) | 143 (66.82) |  |
| AHF, n(%) |  |  |  |  | <.001 |
| No | 1122 (71.88) | 509 (78.43) | 480 (68.77) | 133 (62.15) |  |
| Yes | 439 (28.12) | 140 (21.57) | 218 (31.23) | 81 (37.85) |  |
| Hyperlipidemia, n(%) |  |  |  |  | <.001 |
| No | 1145 (73.35) | 438 (67.49) | 541 (77.51) | 166 (77.57) |  |
| Yes | 416 (26.65) | 211 (32.51) | 157 (22.49) | 48 (22.43) |  |
| Obesity, n(%) |  |  |  |  | <.001 |
| No | 1485 (95.13) | 639 (98.46) | 656 (93.98) | 190 (88.79) |  |
| Yes | 76 (4.87) | 10 (1.54) | 42 (6.02) | 24 (11.21) |  |
| CKD, n(%) |  |  |  |  | <.001 |
| No | 1464 (93.79) | 630 (97.07) | 650 (93.12) | 184 (85.98) |  |
| Yes | 97 (6.21) | 19 (2.93) | 48 (6.88) | 30 (14.02) |  |
| AMI, n(%) |  |  |  |  | 0.303 |
| No | 1445 (92.57) | 593 (91.37) | 653 (93.55) | 199 (92.99) |  |
| Yes | 116 (7.43) | 56 (8.63) | 45 (6.45) | 15 (7.01) |  |
| **Clinical Treatment** |  |  |  |  |  |
| Warfarin, n(%) |  |  |  |  | 0.302 |
| No | 1221 (78.22) | 505 (77.81) | 540 (77.36) | 176 (82.24) |  |
| Yes | 340 (21.78) | 144 (22.19) | 158 (22.64) | 38 (17.76) |  |
| Statin, n(%) |  |  |  |  | 0.102 |
| No | 136 (8.71) | 45 (6.93) | 71 (10.17) | 20 (9.35) |  |
| Yes | 1425 (91.29) | 604 (93.07) | 627 (89.83) | 194 (90.65) |  |
| Beta Blocker, n(%) |  |  |  |  | 0.011 |
| No | 131 (8.39) | 46 (7.09) | 56 (8.02) | 29 (13.55) |  |
| Yes | 1430 (91.61) | 603 (92.91) | 642 (91.98) | 185 (86.45) |  |
| NOAC, n(%) |  |  |  |  | 0.102 |
| No | 1396 (89.43) | 593 (91.37) | 616 (88.25) | 187 (87.38) |  |
| Yes | 165 (10.57) | 56 (8.63) | 82 (11.75) | 27 (12.62) |  |
| Antiplatelet, n(%) |  |  |  |  | 0.234 |
| No | 98 (6.28) | 37 (5.70) | 42 (6.02) | 19 (8.88) |  |
| Yes | 1463 (93.72) | 612 (94.30) | 656 (93.98) | 195 (91.12) |  |
| Metformin, n(%) |  |  |  |  | 0.831 |
| No | 1353 (86.68) | 566 (87.21) | 601 (86.10) | 186 (86.92) |  |
| Yes | 208 (13.32) | 83 (12.79) | 97 (13.90) | 28 (13.08) |  |
| Insulin, n(%) |  |  |  |  | <.001 |
| No | 303 (19.41) | 157 (24.19) | 123 (17.62) | 23 (10.75) |  |
| Yes | 1258 (80.59) | 492 (75.81) | 575 (82.38) | 191 (89.25) |  |
| Vasopressin, n(%) |  |  |  |  | <.001 |
| No | 1421 (91.03) | 607 (93.53) | 633 (90.69) | 181 (84.58) |  |
| Yes | 140 (8.97) | 42 (6.47) | 65 (9.31) | 33 (15.42) |  |
| Octreotide, n(%) |  |  |  |  | 0.261 |
| No | 1554 (99.55) | 648 (99.85) | 693 (99.28) | 213 (99.53) |  |
| Yes | 7 (0.45) | 1 (0.15) | 5 (0.72) | 1 (0.47) |  |
| Ventilated, n(%) |  |  |  |  | 0.076 |
| No | 100 (6.41) | 51 (7.86) | 41 (5.87) | 8 (3.74) |  |
| Yes | 1461 (93.59) | 598 (92.14) | 657 (94.13) | 206 (96.26) |  |
| RRT, n(%) |  |  |  |  | <.001 |
| No | 1351 (86.55) | 607 (93.53) | 589 (84.38) | 155 (72.43) |  |
| Yes | 210 (13.45) | 42 (6.47) | 109 (15.62) | 59 (27.57) |  |
| **Scoring system** |  |  |  |  |  |
| APSIII (scores) | 38.00 (29.00, 52.00) | 35.00 (27.00,48.00) | 39.00 (30.00,51.00) | 46.50 (34.25,60.75) | <.001 |
| OASIS (scores) | 31.00 (26.00, 37.00) | 31.00 (25.00,36.00) | 31.00 (26.00,37.00) | 32.50 (26.00,38.00) | 0.112 |
| LODS (scores) | 4.00 (3.00, 6.00) | 4.00 (2.00,6.00) | 4.00 (3.00,6.00) | 5.00 (3.00,7.00) | <.001 |
| SOFA (scores) | 2.00 (0.00, 3.00) | 2.00 (0.00,3.00) | 2.00 (0.00,3.00) | 2.00 (0.00,4.00) | 0.881 |
| GCS (scores) | 15.00 (15.00, 15.00) | 15.00 (15.00,15.00) | 15.00 (15.00,15.00) | 15.00 (15.00,15.00) | 0.694 |
| **Clinical Outcomes** |  |  |  |  |  |
| Los Icu (days) | 2.22 (1.34, 3.98) | 2.00 (1.27,3.21) | 2.33 (1.45,4.13) | 3.29 (1.77,5.91) | <.001 |
| Los Hospital (days) | 8.09 (5.40, 13.01) | 7.06 (4.97,10.93) | 8.77 (5.72,13.85) | 10.44 (6.65,17.13) | <.001 |
| 30-day mortality, n(%) |  |  |  |  | <.001 |
| No | 1452 (93.02) | 618 (95.22) | 648 (92.84) | 186 (86.92) |  |
| Yes | 109 (6.98) | 31 (4.78) | 50 (7.16) | 28 (13.08) |  |
| 90-day mortality, n(%) |  |  |  |  | <.001 |
| No | 1404 (89.94) | 605 (93.22) | 624 (89.40) | 175 (81.78) |  |
| Yes | 157 (10.06) | 44 (6.78) | 74 (10.60) | 39 (18.22) |  |
| 180-day mortality, n(%) |  |  |  |  | <.001 |
| No | 1351 (86.55) | 591 (91.06) | 596 (85.39) | 164 (76.64) |  |
| Yes | 210 (13.45) | 58 (8.94) | 102 (14.61) | 50 (23.36) |  |
| 365-day mortality, n(%) |  |  |  |  | <.001 |
| No | 1286 (82.38) | 570 (87.83) | 564 (80.80) | 152 (71.03) |  |
| Yes | 275 (17.62) | 79 (12.17) | 134 (19.20) | 62 (28.97) |  |

**Abbreviations**: HR: heart rate; SBP: systolic blood pressure; DBP: diastolic blood pressure; MBP: mean blood pressure; RR: respiratory rate; Spo2: Saturation of Peripheral Oxygen; CRP: C-reactive protein; Bun: blood urea nitrogen; PT: prothrombin time; INR: international normalized ratio; RDW: red blood cell distribution width; eGFR: estimated glomerular filtration rate; WBC: white blood cell; RBC: red blood cell count; ALT: alanine aminotransferase; ALP: aspartate aminotransferase; CTI: C-reactive protein-triglyceride-glucose index; AHF: acute heart failure; CKD: chronic kidney disease; AMI: acute myocardial infarction; NOAC: novel oral anticoagulant; RRT: renal replacement therapy; APSIII: acute physiology score iii; OASIS: oxford acute severity of illness score; LODS: logistic organ dysfunction system; SOFA: sequential organ failure assessment; GCS: glasgow coma scale.

Supplementary Table S2 Baseline characteristics of patients from the eICU-CRD database.

| **Variables** | **Total (n = 242)** | **T1 (n = 112)** | **T2 (n = 117)** | **T3 (n = 13)** | **P value** |
| --- | --- | --- | --- | --- | --- |
| **Demographics** |  |  |  |  |  |
| Age (years) | 67.50 (57.00, 76.75) | 65.50 (55.00,76.25) | 67.00 (57.00,75.00) | 73.00 (71.00,78.00) | 0.200 |
| BMI (kg/m2) | 27.74 (25.97, 29.80) | 27.29 (25.79,28.90) | 28.14 (26.23,30.43) | 29.32 (25.18,31.62) | <.001 |
| Gender, n(%) |  |  |  |  | 0.132 |
| Male | 93 (38.43) | 38 (33.93) | 52 (44.44) | 3 (23.08) |  |
| Female | 149 (61.57) | 74 (66.07) | 65 (55.56) | 10 (76.92) |  |
| Race, n(%) |  |  |  |  | 0.111 |
| White | 174 (71.90) | 83 (74.11) | 85 (72.65) | 6 (46.15) |  |
| Other | 68 (28.10) | 29 (25.89) | 32 (27.35) | 7 (53.85) |  |
| **Vital Signs** |  |  |  |  |  |
| HR (bpm) | 83.79 (75.16, 93.40) | 80.26 (71.53,87.05) | 87.51 (77.77,94.19) | 95.50 (82.19,100.18) | <.001 |
| SBP (mmhg) | 120.96 (109.70, 129.61) | 118.41 (109.03,129.31) | 121.67 (110.51,130.80) | 125.02 (114.99,127.81) | 0.368 |
| DBP (mmhg) | 66.04 (59.35, 72.07) | 67.21 (62.32,72.24) | 64.60 (58.74,71.29) | 62.20 (52.88,67.74) | 0.023 |
| MBP (mmhg) | 80.62 (73.88, 87.48) | 81.19 (75.54,88.25) | 80.26 (72.59,86.27) | 76.47 (67.49,86.27) | 0.413 |
| RR (insp/min) | 19.54 (17.68, 21.81) | 19.19 (17.48,21.16) | 19.67 (17.83,22.32) | 24.13 (21.57,27.10) | <.001 |
| Temperature (℃) | 36.80 (36.60, 37.06) | 36.72 (36.55,36.95) | 36.85 (36.69,37.13) | 36.86 (36.57,37.01) | 0.011 |
| Spo2 (%) | 96.91 (95.76, 98.00) | 97.29 (96.33,98.22) | 96.53 (95.53,97.54) | 96.90 (93.06,97.14) | 0.006 |
| **Laboratory Indicators** |  |  |  |  |  |
| CRP (mg/L) | 10.09 (3.45, 24.26) | 3.40 (1.40,7.11) | 19.55 (10.00,35.62) | 142.00 (129.00,200.04) | <.001 |
| Hematocrit (%) | 31.80 (27.85, 37.55) | 33.73 (28.27,38.92) | 30.66 (26.72,35.00) | 36.00 (32.26,37.49) | 0.015 |
| Platelet (K/μL) | 222.20 (161.71, 264.71) | 215.42 (150.36,264.24) | 226.33 (173.16,263.47) | 192.78 (163.00,286.17) | 0.487 |
| Bun (mg/dL) | 25.07 (16.42, 36.40) | 18.80 (13.49,32.20) | 29.25 (21.00,42.14) | 36.00 (26.33,60.45) | <.001 |
| Creatinine (mg/dL) | 1.17 (0.85, 1.67) | 1.10 (0.82,1.50) | 1.22 (0.86,1.73) | 1.68 (1.42,2.52) | 0.013 |
| Potassium (mEq/L) | 4.00 (3.78, 4.24) | 3.97 (3.75,4.21) | 4.00 (3.80,4.27) | 4.23 (3.73,4.46) | 0.329 |
| Sodium (g/L) | 138.16 (135.91, 140.20) | 138.02 (136.00,140.00) | 138.32 (135.93,140.78) | 136.35 (133.86,141.40) | 0.372 |
| Magnesium (mg/dL) | 1.90 (1.71, 2.10) | 1.90 (1.76,2.07) | 1.88 (1.63,2.10) | 2.16 (2.09,2.22) | 0.001 |
| PT (seconds) | 13.92 (12.22, 16.58) | 13.82 (11.65,15.67) | 13.80 (12.28,16.84) | 17.82 (14.20,19.37) | 0.021 |
| INR | 1.22 (1.10, 1.45) | 1.22 (1.07,1.43) | 1.21 (1.10,1.43) | 1.54 (1.15,1.80) | 0.081 |
| RDW (%) | 15.31 (13.93, 16.79) | 15.07 (13.84,16.74) | 15.40 (14.20,16.98) | 15.41 (14.06,16.20) | 0.648 |
| Bicarbonate (mEq/L) | 25.00 (22.54, 27.00) | 25.50 (23.58,27.00) | 24.43 (22.00,27.33) | 22.20 (21.52,24.14) | 0.005 |
| eGFR (mL/min/1.73m2) | 56.45 (44.12,66.85) | 59.30 (47.55,68.90) | 54.1 (41.95, 63.40) | 57.22 (43.61,65.15) | 0.004 |
| Triglyceride (mg/dl) | 150.66 (109.00, 167.12) | 118.25 (83.75,150.73) | 150.73 (150.50,194.00) | 150.70 (150.68,184.00) | <.001 |
| Fasting blood glucose (mg/dl) | 133.38 (117.72, 162.47) | 123.47 (112.90,151.21) | 135.80 (119.94,171.33) | 180.88 (155.99,216.21) | <.001 |
| WBC (K/uL) | 10.28 (7.74, 14.11) | 9.45 (7.40,12.64) | 10.80 (8.50,15.11) | 17.04 (12.38,20.44) | <.001 |
| RBC (m/uL) | 3.62 (3.12, 4.23) | 3.89 (3.24,4.32) | 3.35 (3.05,4.17) | 3.95 (3.25,4.15) | 0.012 |
| Hemoglobin (g/dL) | 10.40 (9.07, 12.35) | 10.57 (9.24,13.05) | 9.93 (8.90,11.62) | 11.42 (10.64,12.69) | 0.034 |
| ALT (IU/L) | 34.63 (19.75, 57.38) | 30.25 (18.96,49.81) | 38.00 (20.25,62.00) | 44.40 (32.00,97.67) | 0.095 |
| ALP (IU/L) | 89.81 (71.75, 108.56) | 86.96 (67.00,102.35) | 93.33 (74.50,115.00) | 85.78 (67.00,185.11) | 0.253 |
| Total Bilirubin (mg/dL) | 0.70 (0.48, 0.90) | 0.65 (0.46,0.87) | 0.70 (0.47,0.90) | 1.18 (0.85,1.63) | 0.007 |
| Free Calcium (mmol/L) | 8.53 (8.17, 8.89) | 8.61 (8.29,8.95) | 8.47 (8.06,8.82) | 8.33 (7.90,8.44) | 0.059 |
| Phosphate (mg/dL) | 3.31 (2.91, 3.80) | 3.17 (2.84,3.52) | 3.38 (3.04,3.89) | 3.60 (2.80,5.30) | 0.010 |
| Chloride (mEq/L) | 104.00 (101.12, 106.93) | 104.00 (101.00,106.00) | 103.67 (101.57,107.36) | 104.86 (101.29,107.67) | 0.694 |
| Lymphocytes (%) | 13.94 (8.40, 20.96) | 16.00 (9.20,22.00) | 12.84 (8.00,19.23) | 9.67 (8.00,15.10) | 0.070 |
| CTI (index) | 10.13 (9.53, 10.68) | 9.42 (8.93,9.83) | 10.55 (10.31,10.90) | 11.70 (11.61,11.75) | <.001 |
| **Comorbidities** |  |  |  |  |  |
| Hypertension, n(%) |  |  |  |  | 0.211 |
| No | 164 (67.77) | 81 (72.32) | 73 (62.39) | 10 (76.92) |  |
| Yes | 78 (32.23) | 31 (27.68) | 44 (37.61) | 3 (23.08) |  |
| Diabetes, n(%) |  |  |  |  | 0.163 |
| No | 175 (72.31) | 86 (76.79) | 82 (70.09) | 7 (53.85) |  |
| Yes | 67 (27.69) | 26 (23.21) | 35 (29.91) | 6 (46.15) |  |
| AHF, n(%) |  |  |  |  | 0.299 |
| No | 201 (83.06) | 96 (85.71) | 96 (82.05) | 9 (69.23) |  |
| Yes | 41 (16.94) | 16 (14.29) | 21 (17.95) | 4 (30.77) |  |
| Hyperlipidemia, n(%) |  |  |  |  | 0.025 |
| No | 186 (76.86) | 94 (83.93) | 81 (69.23) | 11 (84.62) |  |
| Yes | 56 (23.14) | 18 (16.07) | 36 (30.77) | 2 (15.38) |  |
| Obesity, n(%) |  |  |  |  | 0.179 |
| No | 228 (94.21) | 108 (96.43) | 107 (91.45) | 13 (100.00) |  |
| Yes | 14 (5.79) | 4 (3.57) | 10 (8.55) | 0 (0.00) |  |
| CKD, n(%) |  |  |  |  | 0.069 |
| No | 207 (85.54) | 102 (91.07) | 95 (81.20) | 10 (76.92) |  |
| Yes | 35 (14.46) | 10 (8.93) | 22 (18.80) | 3 (23.08) |  |
| AMI, n(%) |  |  |  |  | 0.217 |
| No | 81 (33.47) | 35 (31.25) | 44 (37.61) | 2 (15.38) |  |
| Yes | 161 (66.53) | 77 (68.75) | 73 (62.39) | 11 (84.62) |  |
| **Clinical Treatment** |  |  |  |  |  |
| Warfarin, n(%) |  |  |  |  | 0.117 |
| No | 213 (88.02) | 94 (83.93) | 106 (90.60) | 13 (100.00) |  |
| Yes | 29 (11.98) | 18 (16.07) | 11 (9.40) | 0 (0.00) |  |
| Statin, n(%) |  |  |  |  | 0.902 |
| No | 197 (81.40) | 91 (81.25) | 96 (82.05) | 10 (76.92) |  |
| Yes | 45 (18.60) | 21 (18.75) | 21 (17.95) | 3 (23.08) |  |
| Beta Blocker, n(%) |  |  |  |  | 0.018 |
| No | 132 (54.55) | 51 (45.54) | 71 (60.68) | 10 (76.92) |  |
| Yes | 110 (45.45) | 61 (54.46) | 46 (39.32) | 3 (23.08) |  |
| Antiplatelet, n(%) |  |  |  |  | 0.721 |
| No | 134 (55.37) | 61 (54.46) | 67 (57.26) | 6 (46.15) |  |
| Yes | 108 (44.63) | 51 (45.54) | 50 (42.74) | 7 (53.85) |  |
| Ventilated, n(%) |  |  |  |  | 0.188 |
| No | 235 (97.11) | 111 (99.11) | 111 (94.87) | 13 (100.00) |  |
| Yes | 7 (2.89) | 1 (0.89) | 6 (5.13) | 0 (0.00) |  |
| Insulin, n(%) |  |  |  |  | 0.094 |
| No | 224 (92.56) | 108 (96.43) | 104 (88.89) | 12 (92.31) |  |
| Yes | 18 (7.44) | 4 (3.57) | 13 (11.11) | 1 (7.69) |  |
| Vasopressin, n(%) |  |  |  |  | 0.008 |
| No | 232 (95.87) | 110 (98.21) | 112 (95.73) | 10 (76.92) |  |
| Yes | 10 (4.13) | 2 (1.79) | 5 (4.27) | 3 (23.08) |  |
| **Scoring system** |  |  |  |  |  |
| OASIS (scores) | 27.00 (21.00, 34.75) | 26.00 (19.00,32.25) | 27.00 (23.00,36.00) | 35.00 (32.00,41.00) | 0.002 |
| APSIII (scores) | 57.00 (38.00, 79.00) | 48.50 (33.75,63.02) | 64.00 (46.00,85.00) | 77.00 (56.00,98.00) | <.001 |
| GCS (scores) | 14.54 (13.33, 15.00) | 14.79 (14.11,15.00) | 14.22 (12.99,15.00) | 11.62 (9.89,13.60) | <.001 |
| **Clinical Outcomes** |  |  |  |  |  |
| Los Icu (days) | 66.50 (36.00, 179.00) | 46.50 (30.75,135.00) | 91.00 (38.00,222.00) | 109.00 (37.00,212.00) | 0.022 |
| 30-day mortality, n(%) |  |  |  |  | <.001 |
| No | 201 (83.06) | 93 (83.04) | 102 (87.18) | 6 (46.15) |  |
| Yes | 41 (16.94) | 19 (16.96) | 15 (12.82) | 7 (53.85) |  |

**Abbreviations**: HR: heart rate; SBP: systolic blood pressure; DBP: diastolic blood pressure; MBP: mean blood pressure; RR: respiratory rate; Spo2: Saturation of Peripheral Oxygen; CRP: C-reactive protein; Bun: blood urea nitrogen; PT: prothrombin time; INR: international normalized ratio; RDW: red blood cell distribution width; eGFR: estimated glomerular filtration rate; WBC: white blood cell; RBC: red blood cell count; ALT: alanine aminotransferase; ALP: aspartate aminotransferase; CTI: C-reactive protein-triglyceride-glucose index; AHF: acute heart failure; CKD: chronic kidney disease; AMI: acute myocardial infarction; APSIII: acute physiology score iii; OASIS: oxford acute severity of illness score; GCS: glasgow coma scale.

Supplementary Table S3 Baseline characteristics of patients from the SRHIP database.

| **Variables** | **Total**  **(n = 105)** | **T1**  **(n = 38)** | **T2**  **(n = 52)** | **T3**  **(n = 15)** | **P**  **value** |
| --- | --- | --- | --- | --- | --- |
| **Demographics** |  |  |  |  |  |
| Age (years) | 72.29 (62.69, 81.01) | 69.12 (63.55,81.97) | 73.19 (62.73,79.49) | 75.32 (61.18,83.24) | 0.962 |
| BMI (kg/m2) | 29.62 (26.40, 32.64) | 28.65 (25.07,30.79) | 30.63 (26.20,33.78) | 31.34 (29.86,35.54) | 0.018 |
| Gender, n(%) |  |  |  |  | 0.097 |
| Male | 68 (64.76) | 21 (55.26) | 34 (65.38) | 13 (86.67) |  |
| Female | 37 (35.24) | 17 (44.74) | 18 (34.62) | 2 (13.33) |  |
| **Vital Signs** |  |  |  |  |  |
| HR (bpm) | 84.68 (76.35, 93.87) | 81.44 (74.52,90.85) | 85.70 (77.50,95.45) | 86.00 (79.29,94.39) | 0.487 |
| SBP (mmhg) | 110.15 (104.24, 119.31) | 110.60 (105.76,120.39) | 111.49 (104.36,123.46) | 109.52 (103.91,116.38) | 0.800 |
| DBP (mmhg) | 60.82 (54.21, 65.14) | 60.38 (54.36,64.34) | 61.59 (55.30,66.68) | 58.10 (52.48,64.50) | 0.554 |
| MBP (mmhg) | 74.84 (70.94, 79.64) | 75.06 (71.10,81.06) | 74.98 (71.11,79.69) | 73.96 (68.44,77.62) | 0.558 |
| RR (insp/min) | 18.88 (17.12, 21.16) | 17.94 (16.85,20.61) | 19.95 (16.99,21.84) | 19.60 (18.18,20.21) | 0.305 |
| Temperature (℃) | 36.80 (36.63, 36.96) | 36.76 (36.47,36.93) | 36.79 (36.65,36.93) | 36.93 (36.87,37.03) | 0.040 |
| Spo2 (%) | 97.34 (96.09, 98.46) | 98.05 (96.89,98.52) | 96.71 (95.65,97.89) | 98.05 (96.31,98.61) | 0.018 |
| **Laboratory Indicators** |  |  |  |  |  |
| CRP (mg/L) | 30.00 (3.00, 90.10) | 2.35 (0.95,4.73) | 49.85 (24.48,91.87) | 169.80 (97.85,196.55) | <.001 |
| Platelet (K/μL) | 210.48 (174.11, 265.32) | 211.98 (182.28,246.92) | 207.48 (177.58,270.05) | 218.51 (132.48,265.91) | 0.798 |
| Bun (mg/dL) | 25.62 (17.43, 39.25) | 22.15 (17.38,32.85) | 26.67 (19.51,33.12) | 39.12 (20.04,50.30) | 0.279 |
| Creatinine (mg/dL) | 1.22 (0.94, 1.64) | 1.03 (0.84,1.47) | 1.26 (1.00,1.73) | 1.47 (1.13,3.40) | 0.033 |
| Potassium (mEq/L) | 4.28 (4.11, 4.48) | 4.34 (4.12,4.43) | 4.23 (4.08,4.55) | 4.34 (4.14,4.52) | 0.918 |
| Sodium (g/L) | 138.49 (136.86, 139.88) | 138.42 (135.87,139.65) | 138.15 (136.90,140.14) | 139.00 (137.73,139.67) | 0.612 |
| PT (seconds) | 13.50 (12.22, 16.84) | 13.27 (12.12,16.01) | 14.86 (12.54,17.31) | 13.12 (12.26,15.82) | 0.229 |
| INR | 1.24 (1.12, 1.54) | 1.19 (1.09,1.46) | 1.33 (1.13,1.58) | 1.20 (1.14,1.46) | 0.181 |
| RDW (%) | 14.81 (13.77, 15.85) | 14.18 (13.49,15.11) | 15.19 (14.19,16.65) | 15.03 (13.60,15.64) | 0.031 |
| Triglyceride (mg/dl) | 136.56 (106.62, 175.93) | 115.61 (85.50,149.35) | 131.79 (116.61,159.39) | 183.56 (167.37,229.55) | <.001 |
| Fasting blood glucose (mg/dl) | 134.00 (121.56, 156.75) | 126.86 (118.26,141.73) | 132.68 (123.38,162.46) | 159.86 (142.10,202.17) | <.001 |
| WBC (K/uL) | 6.00 (2.00, 19.00) | 6.75 (4.00,14.20) | 5.38 (1.67,21.96) | 4.28 (4.00,14.75) | 0.916 |
| RBC (m/uL) | 4.00 (1.75, 12.67) | 4.00 (1.78,8.69) | 3.29 (1.72,17.37) | 7.00 (2.25,16.54) | 0.512 |
| Hemoglobin (g/dL) | 9.82 (8.70, 11.12) | 9.66 (8.35,11.23) | 9.84 (8.81,10.99) | 10.07 (8.70,10.87) | 0.818 |
| ALT (IU/L) | 26.50 (18.43, 45.33) | 26.25 (21.64,39.98) | 27.07 (17.72,51.56) | 24.88 (17.33,31.71) | 0.590 |
| ALP (IU/L) | 91.21 (74.00, 129.57) | 82.61 (72.71,105.22) | 96.58 (76.29,138.16) | 105.00 (68.55,126.75) | 0.148 |
| Phosphate (mg/dL) | 3.65 (3.25, 3.94) | 3.63 (3.36,3.84) | 3.53 (3.24,4.02) | 3.71 (3.40,4.20) | 0.520 |
| Chloride (mEq/L) | 104.00 (102.00, 106.63) | 103.95 (101.37,107.56) | 104.00 (102.08,105.72) | 105.00 (101.92,108.01) | 0.541 |
| Lymphocytes (%) | 19.20 (14.84, 25.48) | 20.06 (16.35,27.56) | 18.76 (14.79,24.34) | 16.96 (12.87,27.47) | 0.041 |
| CTI (index) | 10.53 (9.64, 11.10) | 9.40 (8.92,9.69) | 10.74 (10.48,11.08) | 11.77 (11.55,11.82) | <.001 |
| **Comorbidities** |  |  |  |  |  |
| Hypertension, n(%) |  |  |  |  | 0.568 |
| No | 27 (25.71) | 12 (31.58) | 12 (23.08) | 3 (20.00) |  |
| Yes | 78 (74.29) | 26 (68.42) | 40 (76.92) | 12 (80.00) |  |
| Diabetes, n(%) |  |  |  |  | 0.010 |
| No | 53 (50.48) | 25 (65.79) | 25 (48.08) | 3 (20.00) |  |
| Yes | 52 (49.52) | 13 (34.21) | 27 (51.92) | 12 (80.00) |  |
| CKD, n(%) |  |  |  |  | 0.273 |
| No | 100 (95.24) | 37 (97.37) | 50 (96.15) | 13 (86.67) |  |
| Yes | 5 (4.76) | 1 (2.63) | 2 (3.85) | 2 (13.33) |  |
| **Clinical Treatment** |  |  |  |  |  |
| Warfarin, n(%) |  |  |  |  | 0.455 |
| No | 91 (86.67) | 31 (81.58) | 46 (88.46) | 14 (93.33) |  |
| Yes | 14 (13.33) | 7 (18.42) | 6 (11.54) | 1 (6.67) |  |
| Statin, n(%) |  |  |  |  | 0.399 |
| No | 8 (7.62) | 2 (5.26) | 6 (11.54) | 0 (0.00) |  |
| Yes | 97 (92.38) | 36 (94.74) | 46 (88.46) | 15 (100.00) |  |
| Beta Blocker, n(%) |  |  |  |  | 1.000 |
| No | 13 (12.38) | 5 (13.16) | 6 (11.54) | 2 (13.33) |  |
| Yes | 92 (87.62) | 33 (86.84) | 46 (88.46) | 13 (86.67) |  |
| Antiplatelet, n(%) |  |  |  |  | 0.811 |
| No | 10 (9.52) | 3 (7.89) | 5 (9.62) | 2 (13.33) |  |
| Yes | 95 (90.48) | 35 (92.11) | 47 (90.38) | 13 (86.67) |  |
| Insulin, n(%) |  |  |  |  | 0.164 |
| No | 28 (26.67) | 11 (28.95) | 16 (30.77) | 1 (6.67) |  |
| Yes | 77 (73.33) | 27 (71.05) | 36 (69.23) | 14 (93.33) |  |
| **Scoring system** |  |  |  |  |  |
| APSIII (scores) | 55.00 (45.00, 72.00) | 51.00 (41.00,70.00) | 53.00 (45.00,71.25) | 62.00 (57.50,74.50) | 0.045 |
| GCS (scores) | 15.00 (15.00, 15.00) | 15.00 (15.00,15.00) | 15.00 (15.00,15.00) | 15.00 (15.00,15.00) | 0.993 |
| **Clinical Outcomes** |  |  |  |  |  |
| Los Icu (days) | 4.75 (2.06, 7.24) | 4.53 (1.86,8.83) | 3.73 (1.99,5.67) | 5.73 (5.12,8.43) | 0.031 |
| 30-day mortality, n(%) |  |  |  |  | <.001 |
| No | 84 (80.00) | 31 (81.58) | 43 (82.69) | 10 (66.67) |  |
| Yes | 21 (20.00) | 7 (18.42) | 9 (17.31) | 5 (33.33) |  |

**Abbreviations**: HR: heart rate; SBP: systolic blood pressure; DBP: diastolic blood pressure; MBP: mean blood pressure; RR: respiratory rate; Spo2: Saturation of Peripheral Oxygen; CRP: C-reactive protein; Bun: blood urea nitrogen; PT: prothrombin time; INR: international normalized ratio; RDW: red blood cell distribution width; WBC: white blood cell; RBC: red blood cell count; ALT: alanine aminotransferase; ALP: aspartate aminotransferase; CTI: C-reactive protein-triglyceride-glucose index; CKD: chronic kidney disease; APSIII: acute physiology score iii; GCS: glasgow coma scale.

**Supplementary Table S4** Results of variance inflation factor analysis.

| **Variable Name** | **VIF** |
| --- | --- |
| Octreotide | 1.0537147 |
| Free_Calcium | 1.143332132 |
| ALP | 1.153616364 |
| ALT | 1.158156089 |
| Marriage | 1.170437485 |
| Race | 1.17489157 |
| Ventilated | 1.183035543 |
| RBC | 1.183631385 |
| WBC | 1.187538174 |
| NOAC | 1.190964539 |
| Temperature | 1.214003439 |
| AMI | 1.22889656 |
| Hyperlipidemia | 1.232882522 |
| Total_Bilirubin | 1.236175808 |
| Beta_Blocker | 1.248685261 |
| Statin | 1.251154979 |
| Platelet | 1.25670801 |
| Lymphocytes | 1.276420575 |
| Obesity | 1.293318787 |
| Spo2 | 1.321780859 |
| Antiplatelet | 1.32730916 |
| Potassium | 1.342755003 |
| Magnesium | 1.370081753 |
| Metformin | 1.377053821 |
| AHF | 1.399048768 |
| Hypertension | 1.421653236 |
| BMI | 1.439950936 |
| HR | 1.44167928 |
| Bicarbonate | 1.445396702 |
| Sodium | 1.462685131 |
| RR | 1.47360776 |
| Vasopressin | 1.478081558 |
| SOFA | 1.532969834 |
| RDW | 1.562785704 |
| Warfarin | 1.646777292 |
| Chloride | 1.656142679 |
| GCS | 1.699662864 |
| Insulin | 1.73140882 |
| Diabetes | 1.832234365 |
| Age | 1.93852682 |
| Phosphate | 2.009948974 |
| Los_Hospital | 2.022444977 |
| Los_Icu | 2.044218432 |
| RRT | 2.113443613 |
| OASIS | 2.129586914 |
| Hemoglobin | 2.220580626 |
| CKD | 2.63719108 |
| Hematocrit | 2.723395758 |
| Gender | 2.914425171 |
| LODS | 3.021154838 |
| SBP | 3.115644695 |
| APSIII | 3.451275745 |
| Bun | 3.472392976 |
| CTI | 3.639318576 |

**Abbreviations**: ALP: aspartate aminotransferase; ALT: alanine aminotransferase; RBC: red blood cell count; WBC: white blood cell; NOAC: novel oral anticoagulant; AMI: acute myocardial infarction; Spo2: Saturation of Peripheral Oxygen; AHF: acute heart failure; HR: heart rate; RR: respiratory rate; SOFA: sequential organ failure assessment; RDW: red blood cell distribution width; GCS: glasgow coma scale; RRT: renal replacement therapy; OASIS: oxford acute severity of illness score; CKD: chronic kidney disease; LODS: logistic organ dysfunction system; APSIII: acute physiology score iii; Bun: blood urea nitrogen; CTI: C-reactive protein-triglyceride-glucose index.

**Supplementary Table S5** Results of univariate Cox regression analysis.

| **Variables** | **β** | **S.E** | **Z** | **P** | **HR (95%CI)** |
| --- | --- | --- | --- | --- | --- |
| Octreotide |  |  |  |  |  |
| 0 |  |  |  |  | 1.00 (Reference) |
| 1 | 0.29 | 1.01 | 0.29 | 0.772 | 1.34 (0.19 ~ 9.59) |
| Marriage |  |  |  |  |  |
| 0 |  |  |  |  | 1.00 (Reference) |
| 1 | -0.31 | 0.25 | -1.26 | 0.209 | 0.73 (0.45 ~ 1.19) |
| 2 | 0.01 | 0.24 | 0.05 | 0.960 | 1.01 (0.64 ~ 1.61) |
| Race |  |  |  |  |  |
| 0 |  |  |  |  | 1.00 (Reference) |
| 1 | 0.02 | 0.20 | 0.08 | 0.934 | 1.02 (0.68 ~ 1.51) |
| Ventilated |  |  |  |  |  |
| 0 |  |  |  |  | 1.00 (Reference) |
| 1 | -0.27 | 0.39 | -0.70 | 0.486 | 0.76 (0.35 ~ 1.64) |
| NOAC |  |  |  |  |  |
| 0 |  |  |  |  | 1.00 (Reference) |
| 1 | -0.09 | 0.30 | -0.30 | 0.762 | 0.91 (0.51 ~ 1.63) |
| AMI |  |  |  |  |  |
| 0 |  |  |  |  | 1.00 (Reference) |
| 1 | 0.02 | 0.37 | 0.04 | 0.967 | 1.02 (0.49 ~ 2.09) |
| Hyperlipidemia |  |  |  |  |  |
| 0 |  |  |  |  | 1.00 (Reference) |
| 1 | -1.48 | 0.37 | -4.02 | <.001 | 0.23 (0.11 ~ 0.47) |
| Beta Blocker |  |  |  |  |  |
| 0 |  |  |  |  | 1.00 (Reference) |
| 1 | -1.79 | 0.21 | -8.64 | <.001 | 0.17 (0.11 ~ 0.25) |
| Statin |  |  |  |  |  |
| 0 |  |  |  |  | 1.00 (Reference) |
| 1 | -0.80 | 0.26 | -3.09 | 0.002 | 0.45 (0.27 ~ 0.75) |
| Obesity |  |  |  |  |  |
| 0 |  |  |  |  | 1.00 (Reference) |
| 1 | -0.35 | 0.51 | -0.69 | 0.491 | 0.70 (0.26 ~ 1.91) |
| Antiplatelet |  |  |  |  |  |
| 0 |  |  |  |  | 1.00 (Reference) |
| 1 | -1.13 | 0.26 | -4.36 | <.001 | 0.32 (0.20 ~ 0.54) |
| Metformin |  |  |  |  |  |
| 0 |  |  |  |  | 1.00 (Reference) |
| 1 | -2.61 | 1.00 | -2.60 | 0.009 | 0.07 (0.01 ~ 0.52) |
| AHF |  |  |  |  |  |
| 0 |  |  |  |  | 1.00 (Reference) |
| 1 | 0.24 | 0.20 | 1.24 | 0.214 | 1.28 (0.87 ~ 1.87) |
| Hypertension |  |  |  |  |  |
| 0 |  |  |  |  | 1.00 (Reference) |
| 1 | -0.44 | 0.21 | -2.12 | 0.034 | 0.65 (0.43 ~ 0.97) |
| Vasopressin |  |  |  |  |  |
| 0 |  |  |  |  | 1.00 (Reference) |
| 1 | 1.33 | 0.20 | 6.48 | <.001 | 3.77 (2.53 ~ 5.64) |
| Warfarin |  |  |  |  |  |
| 0 |  |  |  |  | 1.00 (Reference) |
| 1 | -1.21 | 0.32 | -3.79 | <.001 | 0.30 (0.16 ~ 0.56) |
| Insulin |  |  |  |  |  |
| 0 |  |  |  |  | 1.00 (Reference) |
| 1 | -0.90 | 0.20 | -4.44 | <.001 | 0.41 (0.27 ~ 0.60) |
| Diabetes |  |  |  |  |  |
| 0 |  |  |  |  | 1.00 (Reference) |
| 1 | -0.55 | 0.20 | -2.80 | 0.005 | 0.58 (0.39 ~ 0.85) |
| RRT |  |  |  |  |  |
| 0 |  |  |  |  | 1.00 (Reference) |
| 1 | 0.22 | 0.23 | 0.95 | 0.341 | 1.24 (0.80 ~ 1.94) |
| CKD |  |  |  |  |  |
| 0 |  |  |  |  | 1.00 (Reference) |
| 1 | 0.05 | 0.33 | 0.15 | 0.878 | 1.05 (0.55 ~ 2.02) |
| Gender |  |  |  |  |  |
| 0 |  |  |  |  | 1.00 (Reference) |
| 1 | -0.01 | 0.20 | -0.06 | 0.953 | 0.99 (0.67 ~ 1.46) |
| Free Calcium | -4.84 | 1.21 | -3.99 | <.001 | 0.01 (0.00 ~ 0.09) |
| ALP | -0.00 | 0.00 | -0.74 | 0.458 | 1.00 (1.00 ~ 1.00) |
| Platelet | -0.01 | 0.00 | -3.87 | <.001 | 0.99 (0.99 ~ 0.99) |
| ALT | 0.01 | 0.00 | 6.04 | <.001 | 1.01 (1.01 ~ 1.01) |
| RBC | 0.00 | 0.00 | 1.31 | 0.192 | 1.00 (1.00 ~ 1.01) |
| WBC | 0.01 | 0.00 | 2.73 | 0.006 | 1.01 (1.01 ~ 1.01) |
| Temperature | 0.08 | 0.21 | 0.38 | 0.708 | 1.08 (0.72 ~ 1.63) |
| Total Bilirubin | 0.06 | 0.06 | 0.97 | 0.333 | 1.06 (0.94 ~ 1.20) |
| Lymphocytes | -0.04 | 0.01 | -2.90 | 0.004 | 0.96 (0.93 ~ 0.99) |
| Spo2 | -0.12 | 0.03 | -3.86 | <.001 | 0.89 (0.83 ~ 0.94) |
| Potassium | -0.44 | 0.35 | -1.27 | 0.206 | 0.65 (0.33 ~ 1.27) |
| Magnesium | 0.42 | 0.53 | 0.78 | 0.434 | 1.52 (0.53 ~ 4.29) |
| BMI | -0.04 | 0.02 | -2.05 | 0.040 | 0.96 (0.93 ~ 0.99) |
| HR | 0.02 | 0.01 | 2.94 | 0.003 | 1.02 (1.01 ~ 1.03) |
| Bicarbonate | -0.13 | 0.04 | -3.24 | 0.001 | 0.88 (0.81 ~ 0.95) |
| Sodium | 0.08 | 0.03 | 2.37 | 0.018 | 1.08 (1.01 ~ 1.16) |
| RR | 0.13 | 0.02 | 5.48 | <.001 | 1.14 (1.09 ~ 1.20) |
| SOFA | 0.02 | 0.04 | 0.40 | 0.688 | 1.02 (0.94 ~ 1.10) |
| RDW | 0.09 | 0.05 | 1.93 | 0.053 | 1.10 (1.00 ~ 1.20) |
| Chloride | -0.02 | 0.02 | -1.11 | 0.265 | 0.98 (0.94 ~ 1.02) |
| GCS | -0.02 | 0.04 | -0.61 | 0.543 | 0.98 (0.91 ~ 1.05) |
| Age | 0.05 | 0.01 | 5.07 | <.001 | 1.05 (1.03 ~ 1.07) |
| Phosphate | 0.37 | 0.12 | 3.20 | 0.001 | 1.45 (1.15 ~ 1.81) |
| Los Hospital | -0.05 | 0.01 | -3.66 | <.001 | 0.95 (0.92 ~ 0.98) |
| Los Icu | 0.05 | 0.01 | 5.37 | <.001 | 1.04 (1.01 ~ 1.07) |
| OASIS | 0.06 | 0.01 | 5.82 | <.001 | 1.06 (1.03 ~ 1.08) |
| Hemoglobin | 0.04 | 0.06 | 0.64 | 0.521 | 1.04 (0.93 ~ 1.15) |
| CTI | 0.18 | 0.10 | 1.75 | 0.081 | 1.19 (0.98 ~ 1.46) |
| Bun | 0.01 | 0.01 | 2.18 | 0.030 | 1.01 (1.01 ~ 1.02) |
| Hematocrit | -0.00 | 0.02 | -0.19 | 0.847 | 1.00 (0.95 ~ 1.04) |
| LODS | 0.18 | 0.03 | 6.10 | <.001 | 1.19 (1.13 ~ 1.26) |
| SBP | -0.03 | 0.01 | -3.58 | <.001 | 0.97 (0.96 ~ 0.99) |
| APSIII | 0.03 | 0.00 | 7.69 | <.001 | 1.03 (1.02 ~ 1.04) |

**Abbreviations**: NOAC: novel oral anticoagulant; AMI: acute myocardial infarction; AHF: acute heart failure; RRT: renal replacement therapy; CKD: chronic kidney disease; ALP: aspartate aminotransferase; ALT: alanine aminotransferase; RBC: red blood cell count; WBC: white blood cell; Spo2: Saturation of Peripheral Oxygen; HR: heart rate; RR: respiratory rate; SOFA: sequential organ failure assessment; RDW: red blood cell distribution width; GCS: glasgow coma scale; OASIS: oxford acute severity of illness score; CTI: C-reactive protein-triglyceride-glucose index; Bun: blood urea nitrogen; LODS: logistic organ dysfunction system; SBP: systolic blood pressure; APSIII: acute physiology score iii.

**Supplementary Table S6** Mediating effects of disease severity on the association between CTI and all-cause mortality in patients with coronary artery disease

| Mediator | Total effect (95%CI) | P | ACME (95%CI) | P | ADE (95%CI) | P | Proportion Mediated |
| --- | --- | --- | --- | --- | --- | --- | --- |
| **30d** |  |  |  |  |  |  |  |
| APSIII | 0.610 (0.541-0.670) | <0.001 | 0.119 (0.082-0.189) | <0.001 | 0.490 (0.453-0.531) | <0.001 | 19.12% |
| OASIS | 0.607 (0.543-0.671) | <0.001 | 0.017 (0.006-0.031) | <0.001 | 0.591 (0.527-0.641) | <0.001 | 2.77% |
| LODS | 0.610 (0.543-0.668) | <0.001 | 0.071 (0.044-0.116) | <0.001 | 0.539 (0.488-0.579) | <0.001 | 11.13% |
| SOFA | 0.608 (0.547-0.670) | <0.001 | 0.004 (-0.001-0.011) | 0.24 | 0.604 (0.541-0.661) | <0.001 | 0.47% |
| GCS | 0.607 (0.547-0.671) | <0.001 | 0.002 (-0.002-0.007) | 0.56 | 0.606 (0.545-0.668) | <0.001 | 0.26% |
| **90d** |  |  |  |  |  |  |  |
| APSIII | 0.644 (0.454-0.891) | <0.001 | 0.115 (0.064-0.229) | <0.001 | 0.530 (0.390-0.656) | <0.001 | 17.29% |
| OASIS | 0.635 (0.458-0.898) | <0.001 | 0.013 (0.001-0.030) | 0.04 | 0.622 (0.447-0.867) | <0.001 | 2.30% |
| LODS | 0.647 (0.462-0.899) | <0.001 | 0.068 (0.035-0.141) | <0.001 | 0.579 (0.414-0.743) | <0.001 | 10.06% |
| SOFA | 0.634 (0.463-0.904) | <0.001 | 0.001 (-0.003-0.006) | 0.68 | 0.633 (0.462-0.899) | <0.001 | 0.11% |
| GCS | 0.637 (0.470-0.898) | <0.001 | 0.003 (-0.003-0.011) | 0.48 | 0.634 (0.470-0.890) | <0.001 | 0.39% |
| **180d** |  |  |  |  |  |  |  |
| APSIII | 0.606 (0.365-0.980) | <0.001 | 0.103 (0.049-0.242) | <0.001 | 0.502 (0.312-0.723) | <0.001 | 16.39% |
| OASIS | 0.591 (0.367-0.987) | <0.001 | 0.010 (-0.00-0.027) | 0.08 | 0.581 (0.360-0.960) | <0.001 | 1.55% |
| LODS | 0.604 (0.369-0.988) | <0.001 | 0.060 (0.027-0.147) | <0.001 | 0.544 (0.336-0.829) | <0.001 | 9.54% |
| SOFA | 0.589 (0.372-0.995) | <0.001 | 0.001 (-0.000-0.005) | 0.62 | 0.589 (0.372-0.992) | <0.001 | 0.17% |
| GCS | 0.595 (0.382-0.984) | <0.001 | 0.003 (-0.003-0.011) | 0.48 | 0.592 (0.382-0.973) | <0.001 | 0.50% |
| **365d** |  |  |  |  |  |  |  |
| APSIII | 0.544 (0.273-1.039) | <0.001 | 0.101 (0.04-0.274) | <0.001 | 0.443 (0.232-0.741) | <0.001 | 17.59% |
| OASIS | 0.520 (0.273-1.033) | <0.001 | 0.010 (-0.001-0.031) | 0.08 | 0.511 (0.267-1.002) | <0.001 | 1.71% |
| LODS | 0.537 (0.277-1.041) | <0.001 | 0.058 (0.023-0.163) | <0.001 | 0.480 (0.252-0.863) | <0.001 | 10.08% |
| SOFA | 0.519 (0.279-1.045) | <0.001 | 0.001 (-0.000-0.004) | 0.24 | 0.518 (0.278-1.043) | <0.001 | 0.21% |
| GCS | 0.527 (0.289-1.031) | <0.001 | 0.003 (-0.003-0.012) | 0.44 | 0.523 (0.290-1.018) | <0.001 | 0.56% |

Adjusted for Hyperlipidemia, Hypertension, Diabetes, Gender, Spo2, BMI, HR, RR, Age, SBP, Beta Blocker, Statin, Antiplatelet, Metformin, Vasopressin, Warfarin, Insulin, Free Calcium, ALP, ALT, WBC, Platelet, Lymphocytes, Bicarbonate, Sodium, Phosphate, Los Hospital, OASIS, LODS, APSIII, Bun.

**Abbreviations**: HR: Hazard Ratio; CI: Confidence Interval; ACME: average causal mediation effect; ADE: average direct effect; APSIII: acute physiology score iii; OASIS: oxford acute severity of illness score; LODS: logistic organ dysfunction system; SOFA: sequential organ failure assessment; GCS: glasgow coma scale.

**Supplementary Table S7** Sensitivity analysis of exploring the risk association between continuous CTI and CAD.

| Outcome | Model 1, HR (95%CI) | P | Model 2, HR (95%CI) | P | Model 3, HR (95%CI) | P |
| --- | --- | --- | --- | --- | --- | --- |
| **30d** |  |  |  |  |  |  |
| CTI (per 1 unit) | 1.20 (1.08–1.33) | 0.004 | 1.30 (1.15–1.47) | 0.002 | 1.22 (1.05–1.42) | 0.015 |
| **90d** |  |  |  |  |  |  |
| CTI (per 1 unit) | 1.14 (1.03–1.27) | 0.012 | 1.22 (1.10–1.35) | 0.002 | 1.12 (1.01–1.25) | 0.028 |
| **180d** |  |  |  |  |  |  |
| CTI (per 1 unit) | 1.12 (1.02–1.24) | 0.021 | 1.18 (1.07–1.31) | 0.005 | 1.10 (1.00–1.22) | 0.041 |
| **365d** |  |  |  |  |  |  |
| CTI (per 1 unit) | 1.10 (1.01–1.21) | 0.033 | 1.11 (1.03–1.20) | 0.009 | 1.08 (1.00–1.17) | 0.045 |

HR = Hazard Ratio, CI = Confidence Interval

Model 1: unadjusted for any covariates.

Model 2: adjusted for Hyperlipidemia, Hypertension, Diabetes, Gender, Spo2, BMI, HR, RR, Age, SBP.

Model 3: adjusted for Hyperlipidemia, Hypertension, Diabetes, Gender, Spo2, BMI, HR, RR, Age, SBP, Beta Blocker, Statin, Antiplatelet, Metformin, Vasopressin, Warfarin, Insulin, Free Calcium, ALP, ALT, WBC, Platelet, Lymphocytes, Bicarbonate, Sodium, Phosphate, Los Hospital, OASIS, LODS, APSIII, Bun.

**Abbreviations**: Spo2: Saturation of Peripheral Oxygen; HR: heart rate; RR: respiratory rate; SBP: systolic blood pressure; ALP: aspartate aminotransferase; ALT: alanine aminotransferase; WBC: white blood cell; OASIS: oxford acute severity of illness score; LODS: logistic organ dysfunction system; APSIII: acute physiology score iii; Bun: blood urea nitrogen.

**Supplementary Table S8** Sensitivity analysis of the association between CTI and CAD risk using logistic regression.

| Outcome | Model 1, OR (95%CI) | P | Model 2, OR (95%CI) | P | Model 3, OR (95%CI) | P |
| --- | --- | --- | --- | --- | --- | --- |
| **30d** |  |  |  |  |  |  |
| T1 | 1.00 (Reference) |  | 1.00 (Reference) |  | 1.00 (Reference) |  |
| T2 | 1.59 (1.00-2.54) | 0.050 | 1.64 (0.99-2.72) | 0.053 | 1.48 (0.82-2.68) | 0.198 |
| T3 | 3.11 (1.81-5.33) | <.001 | 3.27 (1.77-6.07) | <.001 | 2.77 (1.30-5.91) | 0.008 |
| P for trend | 1.76 (1.33–2.31) | <.001 | 1.97 (1.47–2.62) | <.001 | 1.65 (1.13–2.41 | 0.009 |
| **90d** |  |  |  |  |  |  |
| T1 | 1.00 (Reference) |  | 1.00 (Reference) |  | 1.00 (Reference) |  |
| T2 | 1.61 (1.09-2.37) | 0.017 | 1.69 (1.10-2.58) | 0.017 | 1.39 (0.85-2.26) | 0.190 |
| T3 | 3.06 (1.93-4.87) | <.001 | 3.38 (1.98-5.77) | <.001 | 2.11 (1.12-3.96) | 0.021 |
| P for trend | 1.74 (1.38–2.20) | <.001 | 1.98 (1.55–2.54) | <.001 | 1.45 (1.06–1.98) | 0.022 |
| **180d** |  |  |  |  |  |  |
| T1 | 1.00 (Reference) |  | 1.00 (Reference) |  | 1.00 (Reference) |  |
| T2 | 1.74 (1.24-2.45) | 0.001 | 1.81 (1.24-2.63) | 0.002 | 1.49 (0.99-2.25) | 0.058 |
| T3 | 3.11 (2.05-4.71) | <.001 | 3.46 (2.14-5.58) | <.001 | 2.17 (1.26-3.73) | 0.005 |
| P for trend | 1.76 (1.43–2.17) | <.001 | 2.02 (1.62–2.53) | <.001 | 1.48 (1.13–1.93) | 0.004 |
| **365d** |  |  |  |  |  |  |
| T1 | 1.00 (Reference) |  | 1.00 (Reference) |  | 1.00 (Reference) |  |
| T2 | 1.71 (1.27-2.32) | <.001 | 1.68 (1.21-2.34) | 0.002 | 1.39 (0.96-2.00) | 0.077 |
| T3 | 2.94 (2.02-4.29) | <.001 | 3.05 (1.98-4.72) | <.001 | 1.91 (1.18-3.11) | 0.009 |
| P for trend | 1.72 (1.42–2.07) | <.001 | 1.97 (1.61–2.41) | <.001 | 1.38 (1.09–1.76) | 0.008 |

OR = Odds Ratio, CI = Confidence Interval

Model 1: unadjusted for any covariates.

Model 2: adjusted for Hyperlipidemia, Hypertension, Diabetes, Gender, Spo2, BMI, HR, RR, Age, SBP.

Model 3: adjusted for Hyperlipidemia, Hypertension, Diabetes, Gender, Spo2, BMI, HR, RR, Age, SBP, Beta Blocker, Statin, Antiplatelet, Metformin, Vasopressin, Warfarin, Insulin, Free Calcium, ALP, ALT, WBC, Platelet, Lymphocytes, Bicarbonate, Sodium, Phosphate, Los Hospital, OASIS, LODS, APSIII, Bun.

**Abbreviations**: Spo2: Saturation of Peripheral Oxygen; HR: heart rate; RR: respiratory rate; SBP: systolic blood pressure; ALP: aspartate aminotransferase; ALT: alanine aminotransferase; WBC: white blood cell; OASIS: oxford acute severity of illness score; LODS: logistic organ dysfunction system; APSIII: acute physiology score iii; Bun: blood urea nitrogen.

**Supplementary Table S9** Comparison of the predictive performance of individual indicators.

| **Variable** | **AUC (95%CI)** | **Sensitivity** | **Specificity** |
| --- | --- | --- | --- |
| **30d** |  |  |  |
| TyG | 0.5227 (0.4604 - 0.5833) | 0.3 | 0.82 |
| APSIII | 0.5986 (0.5427 - 0.6592) | 0.61 | 0.56 |
| OASIS | 0.6717 (0.6216 - 0.7212) | 0.79 | 0.48 |
| LODS | 0.7197 (0.6687 - 0.7638) | 0.64 | 0.7 |
| SOFA | 0.5306 (0.4747 - 0.5859) | 0.31 | 0.76 |
| GCS | 0.4020 (0.3584 - 0.4465) | 1 | 0.05 |
| CTI | 0.7892 (0.7527 - 0.8218) | 0.87 | 0.61 |
| CRP | 0.6075 (0.5470 - 0.6690) | 0.58 | 0.62 |
| Triglyceride | 0.4752 (0.4172 - 0.5304) | 1 | 0.03 |
| Glucose | 0.5619 (0.4917 - 0.6311) | 0.38 | 0.82 |
| **90d** |  |  |  |
| TyG | 0.5012 (0.4490 - 0.5575) | 0.28 | 0.82 |
| APSIII | 0.5951 (0.5471 - 0.6445) | 0.72 | 0.43 |
| OASIS | 0.6317 (0.5856 - 0.6777) | 0.74 | 0.48 |
| LODS | 0.6898 (0.6490 - 0.7301) | 0.58 | 0.71 |
| SOFA | 0.5195 (0.4673 - 0.5658) | 0.3 | 0.76 |
| GCS | 0.4143 (0.3793 - 0.4486) | 0.99 | 0.05 |
| CTI | 0.7435 (0.7062 - 0.7768) | 0.8 | 0.59 |
| CRP | 0.6190 (0.5690 - 0.6691) | 0.6 | 0.62 |
| Triglyceride | 0.4608 (0.4142 - 0.5086) | 0.13 | 0.89 |
| Glucose | 0.5569 (0.5007 - 0.6127) | 0.5 | 0.66 |
| **180d** |  |  |  |
| TyG | 0.4989 (0.4501 - 0.5455) | 0.26 | 0.82 |
| APSIII | 0.6030 (0.5604 - 0.6475) | 0.72 | 0.44 |
| OASIS | 0.5991 (0.5576 - 0.6374) | 0.7 | 0.49 |
| LODS | 0.6518 (0.6129 - 0.6904) | 0.52 | 0.71 |
| SOFA | 0.5000 (0.4594 - 0.5391) | 0.28 | 0.76 |
| GCS | 0.4363 (0.4068 - 0.4649) | 0.99 | 0.05 |
| CTI | 0.7036 (0.6684 - 0.7374) | 0.72 | 0.6 |
| CRP | 0.6303 (0.5886 - 0.6722) | 0.64 | 0.6 |
| Triglyceride | 0.4530 (0.4120 - 0.4943) | 0.12 | 0.89 |
| Glucose | 0.5623 (0.5135 - 0.6111) | 0.49 | 0.71 |
| **365-d** |  |  |  |
| TyG | 0.5156 (0.4767 - 0.5568) | 0.38 | 0.72 |
| APSIII | 0.6084 (0.5707 - 0.6444) | 0.79 | 0.38 |
| OASIS | 0.6027 (0.5648 - 0.6387) | 0.68 | 0.49 |
| LODS | 0.6513 (0.6133 - 0.6867) | 0.51 | 0.72 |
| SOFA | 0.4985 (0.4602 - 0.5352) | 0.28 | 0.76 |
| GCS | 0.4501 (0.4251 - 0.4746) | 0.99 | 0.05 |
| CTI | 0.7074 (0.6743 - 0.7366) | 0.72 | 0.61 |
| CRP | 62.43(58.67 - 66.0) | 0.68 | 0.54 |
| Triglyceride | 46.32(42.57 - 50.07) | 1 | 0.01 |
| Glucose | 58.06(53.81 - 62.24) | 0.52 | 0.71 |

CI = Confidence Interval

**Abbreviations**: TyG: triglyceride-glucose index; APSIII: acute physiology score iii; OASIS: oxford acute severity of illness score; LODS: logistic organ dysfunction system; SOFA: sequential organ failure assessment; GCS: glasgow coma scale; CTI: C-reactive protein-triglyceride-glucose index; CRP: C-reactive protein.

**Supplementary Table S10** The DeLong test results of CTI compared with those of other individual indicators.

| **Comparative variable** | **Z statistic** | **P** |
| --- | --- | --- |
| **30d** |  |  |
| TyG | 7.403 | < 0.001 |
| APSIII | 5.489 | < 0.001 |
| OASIS | 5.379 | < 0.001 |
| LODS | 3.308 | < 0.001 |
| SOFA | 8.82 | < 0.001 |
| GCS | 7.836 | < 0.001 |
| CRP | 5.249 | < 0.001 |
| Triglyceride | 8.103 | < 0.001 |
| Glucose | 6.272 | < 0.001 |
| **90d** |  |  |
| TyG | 7.824 | < 0.001 |
| APSIII | 4.889 | < 0.001 |
| OASIS | 5.661 | < 0.001 |
| LODS | 2.951 | 0.00316 |
| SOFA | 8.631 | < 0.001 |
| GCS | 7.284 | < 0.001 |
| CRP | 4.041 | < 0.001 |
| Triglyceride | 6.617 | < 0.001 |
| Glucose | 6.153 | < 0.001 |
| **180d** |  |  |
| TyG | 6.678 | < 0.001 |
| APSIII | 3.753 | < 0.001 |
| OASIS | 5.589 | < 0.001 |
| LODS | 3.259 | 0.00112 |
| SOFA | 8.724 | < 0.001 |
| GCS | 7.253 | < 0.001 |
| CRP | 2.736 | 0.00621 |
| Triglyceride | 5.581 | < 0.001 |
| Glucose | 5.138 | < 0.001 |
| **365d** |  |  |
| TyG | 7.81 | < 0.001 |
| APSIII | 4.273 | < 0.001 |
| OASIS | 5.999 | < 0.001 |
| LODS | 3.882 | < 0.001 |
| SOFA | 7.282 | < 0.001 |
| GCS | 9.11 | < 0.001 |
| CRP | 3.568 | < 0.001 |
| Triglyceride | 6.673 | < 0.001 |
| Glucose | 5.159 | < 0.001 |

**Abbreviations**: TyG: triglyceride-glucose index; APSIII: acute physiology score iii; OASIS: oxford acute severity of illness score; LODS: logistic organ dysfunction system; SOFA: sequential organ failure assessment; GCS: glasgow coma scale; CTI: C-reactive protein-triglyceride-glucose index; CRP: C-reactive protein.

**Supplementary Table S11** Feature selection using Boruta algorithm and LASSO binary logistic regression.

| **Variable Name** | **LASSO** | **Boruta** |
| --- | --- | --- |
| Octreotide |  |  |
| Free_Calcium |  |  |
| ALP | ✓ | ✓ |
| ALT | ✓ |  |
| Marriage | ✓ |  |
| Race |  |  |
| Ventilated |  |  |
| RBC | ✓ | ✓ |
| WBC | ✓ | ✓ |
| NOAC | ✓ |  |
| Temperature |  |  |
| AMI | ✓ |  |
| Hyperlipidemia | ✓ |  |
| Total_Bilirubin | ✓ |  |
| Beta_Blocker | ✓ |  |
| Statin | ✓ |  |
| Platelet | ✓ |  |
| Lymphocytes | ✓ | ✓ |
| Obesity | ✓ |  |
| Spo2 | ✓ |  |
| Antiplatelet |  |  |
| Potassium | ✓ |  |
| Magnesium | ✓ |  |
| Metformin | ✓ |  |
| AHF | ✓ |  |
| Hypertension | ✓ |  |
| BMI | ✓ |  |
| HR | ✓ |  |
| Bicarbonate | ✓ |  |
| Sodium | ✓ | ✓ |
| RR | ✓ | ✓ |
| Vasopressin | ✓ |  |
| SOFA | ✓ |  |
| RDW | ✓ | ✓ |
| Warfarin | ✓ |  |
| Chloride | ✓ | ✓ |
| GCS | ✓ |  |
| Insulin | ✓ |  |
| Diabetes | ✓ |  |
| Age | ✓ | ✓ |
| Phosphate | ✓ | ✓ |
| Los_Hospital | ✓ |  |
| Los_Icu | ✓ | ✓ |
| RRT | ✓ |  |
| OASIS |  |  |
| Hemoglobin |  | ✓ |
| CKD | ✓ |  |
| Hematocrit |  |  |
| Gender | ✓ |  |
| LODS | ✓ |  |
| SBP |  | ✓ |
| APSIII | ✓ | ✓ |
| Bun | ✓ | ✓ |
| CTI | ✓ | ✓ |

**Abbreviations**: ALP: aspartate aminotransferase; ALT: alanine aminotransferase; RBC: red blood cell count; WBC: white blood cell; NOAC: novel oral anticoagulant; AMI: acute myocardial infarction; Spo2: Saturation of Peripheral Oxygen; AHF: acute heart failure; HR: heart rate; RR: respiratory rate; SOFA: sequential organ failure assessment; RDW: red blood cell distribution width; GCS: glasgow coma scale; RRT: renal replacement therapy; OASIS: oxford acute severity of illness score; CKD: chronic kidney disease; LODS: logistic organ dysfunction system; APSIII: acute physiology score iii; Bun: blood urea nitrogen. CTI: C-reactive protein-triglyceride-glucose index.

**Supplementary Table S12** Best hyperparameters of each machine learning model.

| Hyperparameters | LR | KNN | SVC | RF | XGBoost | CNN |
| --- | --- | --- | --- | --- | --- | --- |
| C | 1.5 | – | 2 | – | – | – |
| penalty | 'l2' | – | – | – | – | – |
| solver | 'liblinear' | – | – | – | – | – |
| max_iter | 1000 | – | – | – | – | – |
| class_weight | 'balanced' | – | 'balanced' | 'balanced' | scale_pos_weight='auto' | 'balanced' |
| n_neighbors | – | 21 | – | – | – | – |
| p | – | 1 | – | – | – | – |
| metric | – | 'minkowski' | – | – | – | – |
| weights | – | 'distance' | – | – | – | – |
| kernel | – | – | 'rbf' | – | – | – |
| gamma | – | – | 'scale' | – | 0 | – |
| probability | – | – | TRUE | – | – | – |
| tol | – | – | 0.0001 | – | – | – |
| n_estimators | – | – | – | 300 | 400 | – |
| max_depth | – | – | – | 8 | 5 | – |
| criterion | – | – | – | 'gini' | – | – |
| min_samples_leaf | – | – | – | 20 | – | – |
| min_samples_split | – | – | – | 5 | – | – |
| bootstrap | – | – | – | TRUE | – | – |
| random_state | – | – | – | 42 | – | – |
| learning_rate | – | – | – | – | 0.05 | 0.001 |
| subsample | – | – | – | – | 0.8 | – |
| colsample_bytree | – | – | – | – | 0.8 | – |
| min_child_weight | – | – | – | – | 5 | – |
| reg_lambda | – | – | – | – | 1 | – |
| reg_alpha | – | – | – | – | 0 | – |
| tree_method | – | – | – | – | 'hist' | – |
| eval_metric | – | – | – | – | 'auc' | – |
| conv_blocks | – | – | – | – | – | [(32,3×3),(64,3×3)] |
| pooling | – | – | – | – | – | 'MaxPool(2×2)' |
| dropout | – | – | – | – | – | 0.3 |
| dense_units | – | – | – | – | – | 128 |
| activation | – | – | – | – | – | 'relu' |
| optimizer | – | – | – | – | – | 'adam' |
| batch_size | – | – | – | – | – | 64 |
| epochs | – | – | – | – | – | 50 |
| early_stopping | – | – | – | – | – | True(patience=8) |

**Abbreviations**: LR: logistic regression; KNN: k-nearest neighbor; SVC: support vector classifier; RF: random forest; XGBoost: eXtreme Gradient Boosting; CNN: convolutional neural network.

**Supplementary Table S13** Predictive performance in the internal test set (excluding CTI).

| Model | AUC (95%CI) | F1-score | Accuracy | Precision | Recall |
| --- | --- | --- | --- | --- | --- |
| 30d |  |  |  |  |  |
| LR | 0.8344 (0.7914-0.8747) | 0.7368 | 0.8472 | 0.7292 | 0.7447 |
| KNN | 0.8758 (0.8294-0.9116) | 0.7891 | 0.8917 | 0.7826 | 0.7958 |
| SVC | 0.8724 (0.8334-0.9080) | 0.7826 | 0.8889 | 0.7895 | 0.7759 |
| RF | 0.9175 (0.8878-0.9400) | 0.8525 | 0.9141 | 0.8442 | 0.8609 |
| XGBoost | 0.9124 (0.8820-0.9363) | 0.8428 | 0.9139 | 0.8358 | 0.8499 |
| CNN | 0.9018 (0.8632-0.9324) | 0.7891 | 0.8694 | 0.7651 | 0.8148 |
| 90d |  |  |  |  |  |
| LR | 0.8198 (0.7773-0.8619) | 0.7097 | 0.8361 | 0.7051 | 0.7143 |
| KNN | 0.8816 (0.8525-0.9101) | 0.7785 | 0.8639 | 0.7733 | 0.7838 |
| SVC | 0.8570 (0.8186-0.8929) | 0.7483 | 0.8528 | 0.7432 | 0.7534 |
| RF | 0.9030 (0.8740-0.9293) | 0.8105 | 0.8861 | 0.8052 | 0.8158 |
| XGBoost | 0.8918 (0.8622-0.9197) | 0.8302 | 0.9021 | 0.8246 | 0.8359 |
| CNN | 0.8659 (0.8259-0.9030) | 0.7634 | 0.8556 | 0.7576 | 0.7692 |
| 180d |  |  |  |  |  |
| LR | 0.7863 (0.7329-0.8324) | 0.6552 | 0.7917 | 0.6477 | 0.6629 |
| KNN | 0.8401 (0.7905-0.8810) | 0.7317 | 0.8389 | 0.7229 | 0.7407 |
| SVC | 0.8277 (0.7750-0.8734) | 0.6957 | 0.8194 | 0.6897 | 0.7018 |
| RF | 0.8907 (0.8515-0.9230) | 0.8013 | 0.8806 | 0.7925 | 0.8077 |
| XGBoost | 0.8748 (0.8375-0.9077) | 0.7818 | 0.8694 | 0.7748 | 0.7894 |
| CNN | 0.8405 (0.7942-0.8817) | 0.6885 | 0.8056 | 0.6774 | 0.7001 |
| 365d |  |  |  |  |  |
| LR | 0.8153 (0.7708-0.8535) | 0.6829 | 0.7889 | 0.6747 | 0.6914 |
| KNN | 0.8082 (0.7598-0.8470) | 0.6875 | 0.7917 | 0.6793 | 0.6962 |
| SVC | 0.8406 (0.8022-0.8778) | 0.6933 | 0.8013 | 0.6842 | 0.7027 |
| RF | 0.8919 (0.8603-0.9184) | 0.7586 | 0.8444 | 0.7534 | 0.7639 |
| XGBoost | 0.8817 (0.8490-0.9110) | 0.7919 | 0.8694 | 0.7865 | 0.7975 |
| CNN | 0.8700 (0.8332-0.9040) | 0.7273 | 0.8167 | 0.7164 | 0.7385 |

**Abbreviations**: LR: logistic regression; KNN: k-nearest neighbor; SVC: support vector classifier; RF: random forest; XGBoost: eXtreme Gradient Boosting; CNN: convolutional neural network.

**Supplementary Table S14** Predictive performance in the internal test set (including CTI).

| Model | AUC (95%CI) | F1-score | Accuracy | Precision | Recall | NRI | IDI |
| --- | --- | --- | --- | --- | --- | --- | --- |
| 30d |  |  |  |  |  |  |  |
| LR | 0.8475 (0.8070-0.8862) | 0.7258 | 0.8583 | 0.7155 | 0.7364 | 0.263 | 0.0042 |
| KNN | 0.8945 (0.8505-0.9279) | 0.7812 | 0.8944 | 0.7735 | 0.7891 | -0.0157 | -0.0067 |
| SVC | 0.8939 (0.8594-0.9235) | 0.7965 | 0.8917 | 0.789 | 0.8041 | 0.2728 | 0.0059 |
| RF | 0.9293 (0.8988-0.9525) | 0.8675 | 0.9278 | 0.8592 | 0.8757 | 0.0439 | 0.0005 |
| XGBoost | 0.9190 (0.8862-0.9433) | 0.8354 | 0.9056 | 0.8214 | 0.8499 | 0.1942 | 0.0088 |
| CNN | 0.9122 (0.8736-0.9442) | 0.8146 | 0.8778 | 0.7965 | 0.8336 | 0.1903 | -0.0082 |
| 90d |  |  |  |  |  |  |  |
| LR | 0.8244 (0.7831-0.8647) | 0.7022 | 0.8361 | 0.6948 | 0.7098 | 0.3706 | 0.003 |
| KNN | 0.8890 (0.8582-0.9159) | 0.7819 | 0.8639 | 0.7715 | 0.7928 | 0.0491 | 0.0134 |
| SVC | 0.8688 (0.8334-0.9005) | 0.7514 | 0.8528 | 0.7423 | 0.7611 | 0.2606 | 0.0107 |
| RF | 0.9109 (0.8834-0.9360) | 0.8249 | 0.8987 | 0.8166 | 0.8333 | 0.1847 | 0.0087 |
| XGBoost | 0.8934 (0.8626-0.9209) | 0.8125 | 0.8944 | 0.8025 | 0.8228 | 0.2108 | 0.0082 |
| CNN | 0.8826 (0.8384-0.9220) | 0.7801 | 0.8528 | 0.7688 | 0.7917 | 0.4813 | 0.0116 |
| 180d |  |  |  |  |  |  |  |
| LR | 0.7948 (0.7439-0.8407) | 0.6622 | 0.8056 | 0.654 | 0.6702 | 0.3111 | 0.009 |
| KNN | 0.8156 (0.7617-0.8632) | 0.6944 | 0.8389 | 0.6845 | 0.7047 | -0.1148 | -0.0296 |
| SVC | 0.8366 (0.7874-0.8801) | 0.7248 | 0.8306 | 0.7155 | 0.7343 | 0.2741 | 0.0063 |
| RF | 0.8944 (0.8572-0.9256) | 0.7928 | 0.8778 | 0.7821 | 0.8038 | 0.2296 | 0.0136 |
| XGBoost | 0.8803 (0.8464-0.9130) | 0.7562 | 0.8472 | 0.746 | 0.7667 | 0.0815 | 0.0063 |
| CNN | 0.8662 (0.8216-0.9083) | 0.7188 | 0.8139 | 0.7055 | 0.7325 | -0.1074 | -0.0069 |
| 365d |  |  |  |  |  |  |  |
| LR | 0.8279 (0.7883-0.8650) | 0.6885 | 0.8028 | 0.6795 | 0.6978 | 0.2157 | 0.0072 |
| KNN | 0.8404 (0.7994-0.8774) | 0.7011 | 0.7889 | 0.6912 | 0.7113 | 0.0583 | 0.0183 |
| SVC | 0.8474 (0.8029-0.8856) | 0.7292 | 0.8111 | 0.7179 | 0.7407 | -0.0564 | 0.0058 |
| RF | 0.8969 (0.8632-0.9267) | 0.7815 | 0.8656 | 0.7725 | 0.7907 | 0.0795 | -0.0018 |
| XGBoost | 0.8880 (0.8531-0.9195) | 0.7958 | 0.8778 | 0.7851 | 0.8069 | 0.1884 | 0.0581 |
| CNN | 0.8595 (0.8163-0.8990) | 0.7206 | 0.8083 | 0.7099 | 0.7317 | -0.0018 | 0.0031 |

**Abbreviations**: LR: logistic regression; KNN: k-nearest neighbor; SVC: support vector classifier; RF: random forest; XGBoost: eXtreme Gradient Boosting; CNN: convolutional neural network.

# Supplementary Figure


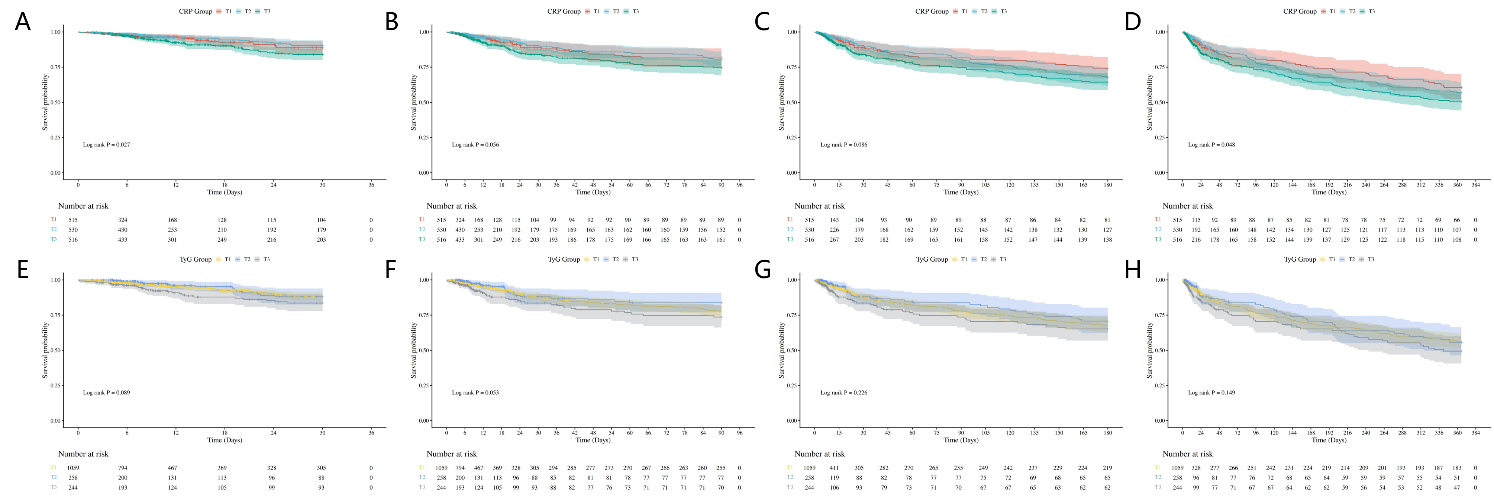


Supplementary Figure 1. The Kaplan-Meier survival curves of CRP and TyG. (A-D) The comparison of all-cause mortality rates within 30 days, 90 days, 180 days, and 365 days for each CRP group. (E-H) The comparison of all-cause mortality rates within 30 days, 90 days, 180 days, and 365 days for each TyG group.

# A representative patient example

To further illustrate the changes in the model's ability to predict patient risk after the inclusion of CTI, this study presents a representative patient case based on a random forest model trained using 30-day all-cause mortality. For this patient, the same set of baseline clinical variables was entered into the RF model without CTI and the CTI-enhanced RF model, respectively, so that the absolute change in predicted mortality risk after incorporation of CTI could be directly compared. The baseline characteristics of the representative patient used for model input are summarized in Table A.

Table A Patient characteristics used for model input

| **Variable** | **Value** |
| --- | --- |
| RDW | 19.58 |
| Bun | 44.4 |
| CTI | 8.991163 |
| ALP | 311 |
| Lymphocytes | 20.33333 |
| Chloride | 102.8 |
| WBC | 7.62 |
| Los_Icu | 41 |
| RBC | 2.85 |
| RR | 17.69333 |
| APSIII | 39 |
| Sodium | 138.4 |
| Platelet | 335.2 |
| Phosphate | 3.864865 |
| Age | 48 |
| Event_30d | 0 |

Using the same patient profile shown in Table A, we further compared the model outputs generated by the RF model without CTI and the RF model with CTI. The resulting predicted risks and classification outputs are presented in Table B.

Table B Predicted risk comparison

| **Model** | **Predicted 30-day mortality risk** | **Binary classification** |
| --- | --- | --- |
| RF model without CTI | 14.5% | Survive |
| RF model with CTI | 9.5% | Survive |

This representative example was intended to demonstrate that the contribution of CTI was reflected not only in overall model discrimination and reclassification performance, but also in individualized risk estimation. In this case, the patient’s true 30-day outcome was survival. The RF model without CTI predicted a 30-day mortality risk of 14.5%, whereas the CTI-enhanced RF model predicted a lower mortality risk of 9.5%, representing an absolute reduction of 5.0 percentage points. Although the binary classification remained unchanged (“Survive”), the prediction after incorporation of CTI was more consistent with the patient’s actual outcome, suggesting that incorporation of CTI may optimize individualized risk estimation for certain patients beyond dichotomous classification alone.

This representative example visually demonstrates how the individualized predicted risk for patients changes after the incorporation of CTI into the machine learning model. However, this example is only for illustrative purposes, but it helps to showcase the potential clinical significance of integrating CTI into predictive models.

# Assessment of patient selection bias

To assess the potential selection bias introduced by restricting the analysis to patients with complete CTI components, we compared the baseline characteristics and crude outcomes between the included patients and those excluded because of missing CTI-related variables in table C.

Table C Baseline comparison between included patients and excluded patients

| **Variables** | **Exclude patients (n = 7578)** | **Included patients (n = 1561)** | **P** |
| --- | --- | --- | --- |
| **Demographics** |  |  |  |
| Age (years) | 70.27 (62.20, 78.05) | 70.19 (61.74, 77.82) | 0.211 |
| BMI (kg/m2) | 28.95 (25.64, 32.84) | 29.48 (26.03, 33.95) | <.001 |
| Gender, n(%) |  |  | <.001 |
| Male | 5553 (73.28) | 1015 (65.02) |  |
| Female | 2025 (26.72) | 546 (34.98) |  |
| Marriage, n(%) |  |  | <.001 |
| Married | 4192 (62.77) | 860 (57.76) |  |
| Single | 1186 (17.76) | 334 (22.43) |  |
| Other | 1300 (19.47) | 295 (19.81) |  |
| Race, n(%) |  |  | 0.007 |
| White | 4735 (62.48) | 1032 (66.11) |  |
| Other | 2843 (37.52) | 529 (33.89) |  |
| **Vital Signs** |  |  |  |
| HR (bpm) | 80.60 (73.77, 88.27) | 81.40 (74.31, 90.49) | <.001 |
| SBP (mmhg) | 112.20 (106.20, 119.09) | 113.07 (106.44, 121.25) | 0.003 |
| DBP (mmhg) | 58.86 (54.00, 64.54) | 59.21 (53.97, 66.20) | 0.013 |
| MBP (mmhg) | 75.12 (70.92, 80.08) | 75.19 (70.69, 81.25) | 0.218 |
| RR (insp/min) | 18.03 (16.35, 20.04) | 18.42 (16.70, 20.64) | <.001 |
| Temperature (℃) | 36.73 (36.54, 36.93) | 36.75 (36.56, 36.98) | 0.003 |
| Spo2 (%) | 97.50 (96.32, 98.54) | 97.34 (96.13, 98.48) | 0.005 |
| **Laboratory Indicators** |  |  |  |
| Hematocrit (%) | 32.10 (29.35, 35.37) | 32.39 (29.33, 35.85) | 0.111 |
| Platelet (K/μL) | 194.90 (158.89, 238.25) | 225.63 (183.13, 274.26) | <.001 |
| Bun (mg/dL) | 19.93 (15.62, 28.40) | 23.07 (17.13, 36.19) | <.001 |
| Creatinine (mg/dL) | 1.00 (0.83, 1.34) | 1.14 (0.89, 1.65) | <.001 |
| Potassium (mEq/L) | 4.22 (4.06, 4.39) | 4.28 (4.10, 4.46) | <.001 |
| Sodium (g/L) | 138.17 (136.55, 139.67) | 138.44 (136.77, 139.90) | 0.001 |
| Magnesium (mg/dL) | 2.12 (2.01, 2.25) | 2.07 (1.97, 2.19) | <.001 |
| PT (seconds) | 13.62 (12.63, 15.14) | 13.82 (12.56, 16.78) | <.001 |
| INR | 1.23 (1.15, 1.38) | 1.26 (1.14, 1.54) | <.001 |
| RDW (%) | 13.90 (13.19, 14.97) | 14.72 (13.71, 16.01) | <.001 |
| Bicarbonate (mEq/L) | 25.11 (23.55, 26.67) | 24.97 (23.52, 26.48) | 0.294 |
| eGFR (mL/min/1.73m2) | 59.59 (49.72, 67.18) | 55.78 (43.80, 65.30) | <.001 |
| Triglyceride (mg/day) | 118.71 (85.00, 169.00) | 131.80 (95.00, 183.86) | <.001 |
| Glucose (mmol/l) | 130.95 (120.41, 147.06) | 134.29 (121.20, 160.35) | <.001 |
| WBC (K/uL) | 4.00 (1.00, 14.00) | 5.56 (2.00, 16.75) | <.001 |
| RBC (m/uL) | 3.00 (1.00, 10.00) | 3.85 (1.53, 10.21) | <.001 |
| Hemoglobin (g/dL) | 10.33 (9.10, 11.50) | 9.80 (8.75, 11.15) | <.001 |
| ALT (IU/L) | 25.00 (18.00, 39.75) | 25.93 (18.42, 44.73) | 0.016 |
| ALP (IU/L) | 74.00 (59.50, 95.00) | 90.00 (70.70, 118.56) | <.001 |
| Total Bilirubin (mg/dL) | 0.55 (0.40, 0.80) | 0.50 (0.37, 0.72) | <.001 |
| Free Calcium (mmol/L) | 1.14 (1.11, 1.18) | 1.14 (1.11, 1.18) | 0.042 |
| Phosphate (mg/dL) | 3.40 (3.03, 3.81) | 3.54 (3.22, 3.98) | <.001 |
| Chloride (mEq/L) | 105.50 (103.00, 107.50) | 105.00 (102.33, 107.00) | <.001 |
| Lymphocytes (%) | 17.00 (11.80, 23.20) | 17.88 (12.99, 23.42) | <.001 |
| **Comorbidities** |  |  |  |
| Hypertension, n(%) |  |  | 0.437 |
| No | 1713 (22.60) | 367 (23.51) |  |
| Yes | 5865 (77.40) | 1194 (76.49) |  |
| Diabetes, n(%) |  |  | <.001 |
| No | 4677 (61.72) | 796 (50.99) |  |
| Yes | 2901 (38.28) | 765 (49.01) |  |
| AHF, n(%) |  |  | <.001 |
| No | 6087 (80.32) | 1122 (71.88) |  |
| Yes | 1491 (19.68) | 439 (28.12) |  |
| Hyperlipidemia, n(%) |  |  | 0.025 |
| No | 5345 (70.53) | 1145 (73.35) |  |
| Yes | 2233 (29.47) | 416 (26.65) |  |
| Obesity, n(%) |  |  | <.001 |
| No | 7374 (97.31) | 1485 (95.13) |  |
| Yes | 204 (2.69) | 76 (4.87) |  |
| CKD, n(%) |  |  | <.001 |
| No | 7352 (97.02) | 1464 (93.79) |  |
| Yes | 226 (2.98) | 97 (6.21) |  |
| AMI, n(%) |  |  | 0.164 |
| No | 6934 (91.50) | 1445 (92.57) |  |
| Yes | 644 (8.50) | 116 (7.43) |  |
| **Clinical Treatment** |  |  |  |
| Warfarin, n(%) |  |  | 0.023 |
| No | 6118 (80.73) | 1221 (78.22) |  |
| Yes | 1460 (19.27) | 340 (21.78) |  |
| Statin, n(%) |  |  | 0.001 |
| No | 489 (6.45) | 136 (8.71) |  |
| Yes | 7089 (93.55) | 1425 (91.29) |  |
| Beta Blocker, n(%) |  |  | 0.024 |
| No | 514 (6.78) | 131 (8.39) |  |
| Yes | 7064 (93.22) | 1430 (91.61) |  |
| NOAC, n(%) |  |  | <.001 |
| No | 7034 (92.82) | 1396 (89.43) |  |
| Yes | 544 (7.18) | 165 (10.57) |  |
| Antiplatelet, n(%) |  |  | <.001 |
| No | 301 (3.97) | 98 (6.28) |  |
| Yes | 7277 (96.03) | 1463 (93.72) |  |
| Metformin, n(%) |  |  | 0.380 |
| No | 6504 (85.83) | 1353 (86.68) |  |
| Yes | 1074 (14.17) | 208 (13.32) |  |
| Insulin, n(%) |  |  | 0.225 |
| No | 1372 (18.11) | 303 (19.41) |  |
| Yes | 6206 (81.89) | 1258 (80.59) |  |
| Vasopressin, n(%) |  |  | 0.093 |
| No | 6994 (92.29) | 1421 (91.03) |  |
| Yes | 584 (7.71) | 140 (8.97) |  |
| Octreotide, n(%) |  |  | 0.889 |
| No | 7542 (99.52) | 1554 (99.55) |  |
| Yes | 36 (0.48) | 7 (0.45) |  |
| Ventilated, n(%) |  |  | 0.518 |
| No | 453 (5.98) | 100 (6.41) |  |
| Yes | 7125 (94.02) | 1461 (93.59) |  |
| RRT, n(%) |  |  | <.001 |
| No | 7142 (94.25) | 1351 (86.55) |  |
| Yes | 436 (5.75) | 210 (13.45) |  |
| **Scoring system** |  |  |  |
| APSIII (scores) | 35.00 (27.00, 48.00) | 38.00 (29.00, 52.00) | <.001 |
| OASIS (scores) | 32.00 (26.00, 37.00) | 31.00 (26.00, 37.00) | 0.109 |
| LODS (scores) | 4.00 (3.00, 6.00) | 4.00 (3.00, 6.00) | 0.006 |
| SOFA (scores) | 2.00 (0.00, 4.00) | 2.00 (0.00, 3.00) | 0.460 |
| GCS (scores) | 15.00 (15.00, 15.00) | 15.00 (15.00, 15.00) | 0.467 |
| **Clinical Outcomes** |  |  |  |
| Los Icu (days) | 2.05 (1.28, 3.30) | 2.22 (1.34, 3.98) | <.001 |
| Los Hospital (days) | 6.87 (4.78, 9.97) | 8.09 (5.40, 13.01) | <.001 |
| 30-day mortality, n(%) |  |  | 0.038 |
| No | 6928 (91.42) | 1452 (93.02) |  |
| Yes | 650 (8.58) | 109 (6.98) |  |
| 90-day mortality, n(%) |  |  | 0.169 |
| No | 6725 (88.74) | 1404 (89.94) |  |
| Yes | 853 (11.26) | 157 (10.06) |  |
| 180-day mortality, n(%) |  |  | 0.539 |
| No | 6602 (87.12) | 1351 (86.55) |  |
| Yes | 976 (12.88) | 210 (13.45) |  |
| 365-day mortality, n(%) |  |  | 0.030 |
| No | 6410 (84.59) | 1286 (82.38) |  |
| Yes | 1168 (15.41) | 275 (17.62) |  |

The comparison showed that some baseline characteristics differed between the two groups, particularly with respect to comorbidity burden, renal function–related indicators, several laboratory parameters, illness severity, and treatment intensity. These findings suggest that the final analytic cohort may have preferentially included patients who underwent more complete laboratory evaluation and had relatively more complex clinical conditions. Therefore, a certain degree of selection bias cannot be fully excluded.

However, this issue mainly reflects the inherent constraints of retrospective database research and the data availability required for CTI calculation, rather than subjective selection during cohort construction. Because CTI was derived from CRP, triglycerides, and glucose, complete measurements of all three components were necessary for its calculation, making this exclusion process methodologically unavoidable. In addition, the crude mortality differences between the included and excluded groups were not uniformly in the same direction across follow-up periods, suggesting that the observed differences may reflect variations in testing patterns and clinical profiles rather than a simple selection of more severely ill patients. Overall, these results indicate that potential selection bias should be acknowledged when interpreting the findings, although the cohort selection was based on objective data completeness criteria.
