# Supplementary figures and images for: Development and validation of the C-reactive protein–triglyceride-glucose index for predicting short- and long-term mortality in critically ill patients with coronary artery disease: a multicenter cohort study
Source: Front Cardiovasc Med. 2026 May 13;13:1763569. doi: 10.3389/fcvm.2026.1763569 (PMC13216771; doi:10.3389/fcvm.2026.1763569)

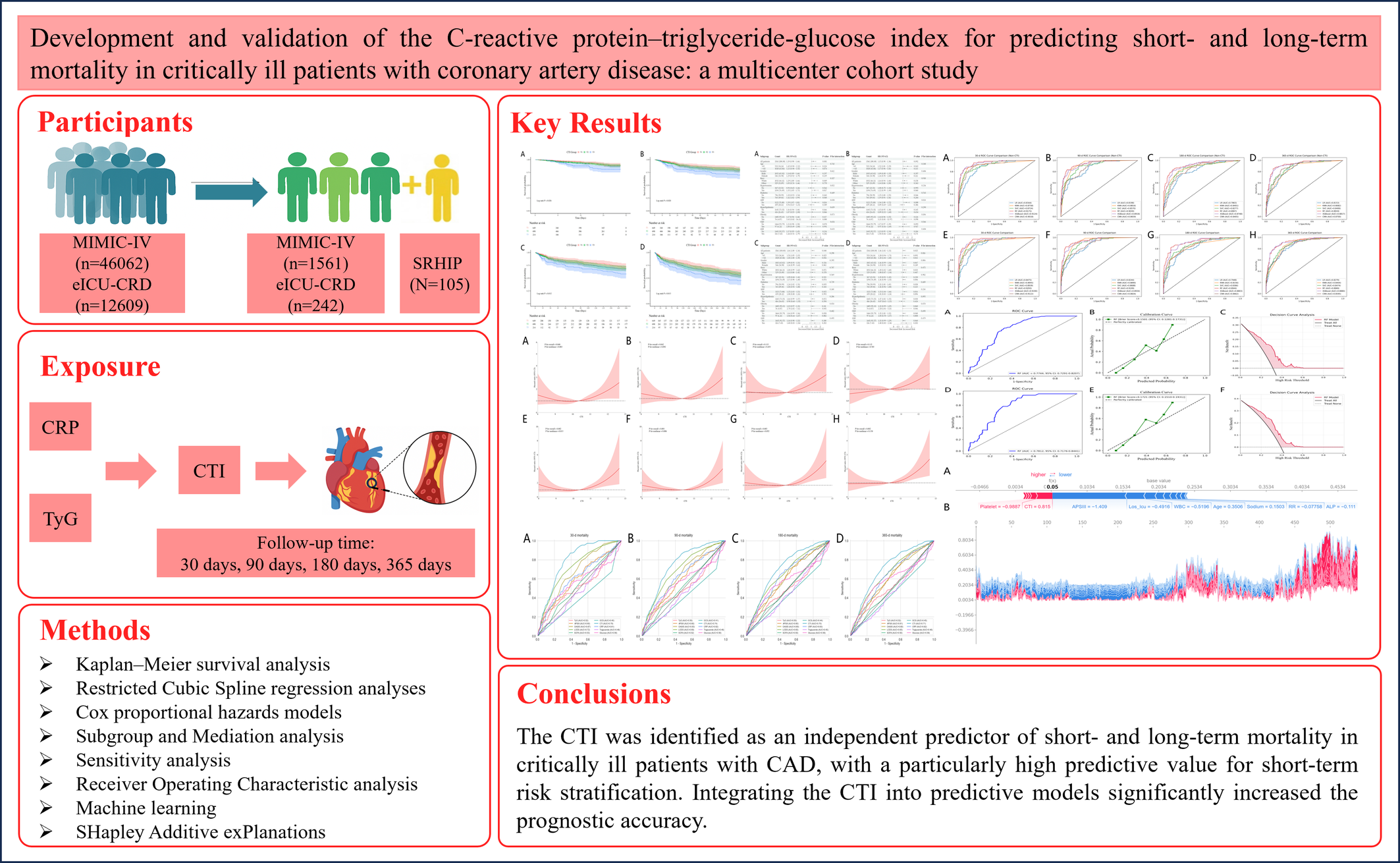

Supplement: Supplementary file 2 [file Image1.tif]
